# Age‐related changes to adipose tissue and peripheral neuropathy in genetically diverse HET3 mice differ by sex and are not mitigated by rapamycin longevity treatment

**DOI:** 10.1111/acel.13784

**Published:** 2023-02-16

**Authors:** Jake W. Willows, Morganne Robinson, Zahra Alshahal, Samantha K. Morrison, Gargi Mishra, Harrison Cyr, Magdalena Blaszkiewicz, Gilian Gunsch, Sabrina DiPietro, Emma Paradie, Benjamin Tero, Anne Harrington, Larisa Ryzhova, Lucy Liaw, Peter C. Reifsnyder, David E. Harrison, Kristy L. Townsend

**Affiliations:** ^1^ Department of Neurological Surgery The Ohio State University, Wexner Medical Center Columbus Ohio USA; ^2^ University of Maine Orono Maine USA; ^3^ Maine Medical Center Research Institute Scarborough Maine USA; ^4^ The Jackson Laboratory Bar Harbor Maine USA

**Keywords:** adipose tissue, aging, collagen, fibrosis, HET3 mice, neuromuscular junction (NMJ), peripheral neuropathy, rapamycin, sex differences

## Abstract

Neural communication between the brain and adipose tissues regulates energy expenditure and metabolism through modulation of adipose tissue functions. We have recently demonstrated that under pathophysiological conditions (obesity, diabetes, and aging), total subcutaneous white adipose tissue (scWAT) innervation is decreased (‘adipose neuropathy’). With advanced age in the C57BL/6J mouse, small fiber peripheral nerve endings in adipose tissue die back, resulting in reduced contact with adipose‐resident blood vessels and other cells. This vascular neuropathy and parenchymal neuropathy together likely pose a physiological challenge for tissue function. In the current work, we used the genetically diverse HET3 mouse model to investigate the incidence of peripheral neuropathy and adipose tissue dysregulation across several ages in both male and female mice. We also investigated the anti‐aging treatment rapamycin, an mTOR inhibitor, as a means to prevent or reduce adipose neuropathy. We found that HET3 mice displayed a reduced neuropathy phenotype compared to inbred C56BL/6 J mice, indicating genetic contributions to this aging phenotype. Compared to female HET3 mice, male HET3 mice had worse neuropathic phenotypes by 62 weeks of age. Female HET3 mice appeared to have increased protection from neuropathy until advanced age (126 weeks), after reproductive senescence. We found that rapamycin overall had little impact on neuropathy measures, and actually worsened adipose tissue inflammation and fibrosis. Despite its success as a longevity treatment in mice, higher doses and longer delivery paradigms for rapamycin may lead to a disconnect between life span and beneficial health outcomes.

## Graphical abstract

Genetically diverse HET3 mice display sex‐dependent peripheral neuropathy phenotypes as a result of age. Longevity treatment rapamycin was unable to attenuate denervation and worsened adipose tissue inflammation and fibrosis.

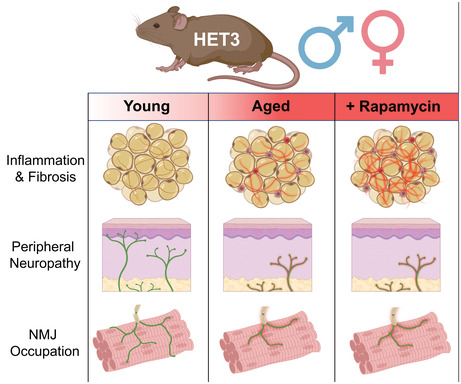

## 1 INTRODUCTION

Aging is the predominant risk factor for disease and is associated with numerous comorbidities that are linked to peripheral nervous system function and metabolic control (Niccoli & Partridge, 2012). For example, with aging, there is increased incidence of peripheral neuropathy (Brisset & Nicolas, 2018), cardiovascular disease (North & Sinclair, 2012), and type 2 diabetes mellitus (Kirkman et al., 2012). With aging, there is also a redistribution of adipose tissue with more ectopic lipid deposition in heart, muscle, and liver, as well as reduced capacity for browning (development of uncoupling protein 1 (UCP1)‐expressing brown adipocytes in white adipose tissue (WAT) depots), and increased inflammation of adipose tissues (Palmer & Kirkland, 2016). WAT is a highly plastic organ, capable of remodeling and changing tissue composition in response to metabolic demands. For example, the tissue can increase cell size or cell number (hypertrophy and hyperplasia, respectively) and initiate browning in response to various stimuli such as cold stimulation (a process that requires mitochondrial biogenesis and neurovascular remodeling). Therefore, aging may represent a loss of these remodeling capabilities, driven by pathophysiological changes to the tissue such as inflammation, fibrosis, and neuropathy (Blaszkiewicz et al., 2019; Khan et al., 2009; Palmer & Kirkland, 2016).

Loss of proper tissue and organ innervation with aging‐related peripheral neuropathy may also underlie many of the observed phenotypes of aging, since the nervous system is known to regulate adipose tissue lipolysis, muscle function, and other metabolically relevant processes. Differences in numerous comorbidities of aging are known to differ by sex and by genetics (Karastergiou et al., 2012; Lumish et al., 2020; Varghese et al., 2020). Sex differences are also important when investigating adipose dysfunction as females display protection from weight gain, insulin resistance, macrophage infiltration, fibrosis, and cell death with a high‐fat diet (Chang et al., 2018).

For this study, we used male and female HET3 mice; the genetically diverse mouse strain utilized by the National Institute on Aging (NIA)'s Interventions Testing Program (ITP; Miller et al., 2007). The HET3 mouse population is produced by crossing BALB/cJ × C57BL/6J F1 females mated to C3H/HeJ × DBA/2 J F1 males. This four‐way cross of inbred strains creates reproducible genetic variability in the offspring (Miller et al., 1999), and age‐related mortality will therefore not be the result of a strain‐specific disease (Flurkey et al., 2010), making HET3 a more relevant model for investigating human aging pathophysiology. Importantly, the average life span of HET3 mice is approximately 120 weeks, which is the same as the inbred C57BL/6J (BL6) strain used as a reference in this study (Flurkey et al., 2010; Yuan et al., 2012).

To date, the ITP has tested over 50 treatments to assess improvements to longevity in the HET3 mouse, across three testing sites (The Jackson Laboratory, The University of Michigan, and the University of Texas Health Science Center), and five of these treatments were moved to a second phase of testing due to effectiveness at extending mean life span; one of which was rapamycin. Rapamycin increased life span of both male and female HET3 mice, regardless of starting the treatment early (Miller et al., 2011) or late in life (Harrison et al., 2009). (*Of note*, *the majority of other ITP‐tested longevity candidate treatments were only effective in expanding life span in males*, *also underscoring the striking sex differences in aging processes*.)

Rapamycin is an inhibitor of the mechanistic target of rapamycin (mTOR) protein which is an integral component in several pathways that govern cell survival, cell proliferation, lipid metabolism, lipid synthesis, and adipogenesis, among many others (Laplante & Sabatini, 2009). mTOR functions as the catalytic component of two protein complexes: rapamycin‐sensitive mTORC1 and rapamycin‐insensitive mTORC2 (Laplante & Sabatini, 2009). Acute rapamycin treatment inhibits mTORC1 promoting increased life span, but chronic (on the order of weeks to months) rapamycin treatment can inhibit mTORC2 in addition to its main target of mTORC1, leading to glucose intolerance (Lamming et al., 2013), delayed glucose clearance (Reifsnyder et al., 2020), insulin insensitivity in BL6 (Lamming et al., 2012) but not HET3 mice (Lamming et al., 2013), hyperlipidemia, and reduced browning‐potential of WAT (Tran et al., 2016). Interestingly, these are also all characteristics of advanced age (DeFronzo, 1981; Palmer & Kirkland, 2016; Rosada et al., 2020). A comprehensive set of experiments were carried out to see if rapamycin had any effect on several structural and functional aging phenotypes in BL6 mice (Neff et al., 2013), which revealed that while rapamycin did increase mean life span, only a handful of the aging phenotypes were improved, and these were also improved in young mice. This suggested that longevity effects were dissociated from effects on aging pathophysiology (Neff et al., 2013). Rapamycin has been shown to be ineffective at increasing the life span of some mouse strains with pre‐existing metabolic disease, resulting in decreased mean life span (Selvarani et al., 2021). These include the obese and diabetic C57BL/KsJ*lepr* ^*db/db*^ mice (Reifsnyder et al., 2018; Sataranatarajan et al., 2016), though there were clear cardioprotective effects in this strain after treatment (Reifsnyder et al., 2018).

Chronic rapamycin treatment has been shown to improve age‐related learning and memory deficits in rodents (Neff et al., 2013) by attenuating the loss in synaptic density driven by mTOR (Van Skike et al., 2020). This poses an intriguing dichotomy in which chronic rapamycin treatment can potentially restore nerve function centrally, while simultaneously not having any protective effects on peripheral nerve health, potentially indirectly by exacerbating diabetic status. Until now, this rapamycin longevity treatment had not yet been comprehensively investigated in adipose tissue function and peripheral neuropathy in the HET3 mouse.

For these reasons, we utilized the 42 ppm encapsulated rapamycin treatment in male and female HET3 mice (previously shown to increase the life span of both sexes by 23%–26% [Miller et al., 2014]) for a chronic 8‐month intervention commenced at two different ages (creating early‐ and late‐intervention groups) to investigate whether rapamycin treatment would attenuate or accelerate age‐related neuropathy in scWAT and other peripheral tissues such as skin and muscle. Age‐related impacts on adipose tissue health and peripheral neuropathy were not mitigated by rapamycin treatment, and adipose inflammation fibrosis was actually worsened after treatment, further underscoring the dissociation between life span and health outcomes of this longevity treatment.

## 2 METHODS

### 2.1 Animals

Female CByB6F1/J (JAX® #100009) bred to male C3D2F1/J (JAX® #100004) at the Jackson Laboratory to produce the male and female HET3 offspring used in this study. Male C57BL/6J (BL6) (JAX® #000664) were also bred and housed at the Jackson Laboratory in the same room as the HET3 mice to limit confounding variables. Mice were housed initially as 4 to a cage in a climate‐controlled vivarium with 12/12 h light/dark cycle and ad libitum access to food and water. Mice were euthanized by CO_2_ asphyxiation with cervical dislocation as a secondary confirmation of death. All procedures were performed in compliance with the National Institute of Health Guide for the Care and Use of Laboratory Animals and was approved by an Institutional Animal Care and Use Committee.

### 2.2 Rapamycin treatment

Forty‐two ppm rapamycin was microencapsulated in chow (LabDiet® 5LG6) and fed to male and female HET3 mice for 8 months prior to behavioral tests and tissue collection. Control mice not receiving rapamycin treatment received standard chow (LabDiet® 5LG6). All mice were fed ad libitum. Mice began rapamycin treatment at one of two ages. Mice that started rapamycin treatment at ~30 weeks of age served as the early‐intervention group, and mice that started treatment at ~72 weeks of age served as the late‐intervention group.

### 2.3 Manual Von Frey tactile sensitivity and nociception test

Manual von Frey tactile sensitivity and nociception test were performed on BL6 and HET3 mice of various ages to determine tactile sensitivity of hind paw skin. Mice were acclimated on top of a grid platform in individual clear‐walled compartments for 1 h. Following acclimation period, von Frey monofilaments were applied to mouse hind paw in order of decreasing strength (4.00 g, 2.00 g, 1.00 g, 0.40 g, and 0.02 g) (Stoelting, Cat#58011), repeated five times for each filament with a 5‐min interval between subsequent pokes on the same mouse. Each filament was applied to the mid‐plantar surface of the hind paw and slight pressure applied until the mouse showed a response or the filament bent with force. A positive response was determined as immediate (<1 s) withdrawal or licking of the paw.

### 2.4 Hematoxylin staining and cell size quantification

Intact inguinal scWAT (ing‐scWAT), axillary scWAT (ax‐scWAT), and perigonadal WAT (pgWAT) depots were excised from BL6 and HET3 mice, fixed in 10% buffered formalin overnight at room temperature and embedded in paraffin. Paraffin‐embedded tissues were sectioned 7 μm thick. Tissue sections were deparaffinized with HistoChoice Clearing Agent and hydrated in decreasing concentrations of EtOH (100%, 95% 70%, 30%, 0%). Tissues were stained in Mayer's Hematoxylin Solution (Sigma‐Aldrich Cat#MHS16) or Mayer's Hemalum Solution (Sigma‐Aldrich Cat#109249) and a drop of mounting fluid was applied, and tissues were cover slipped, sealed, and imaged. Three representative images were captured per tissue per animal and cell perimeter and area were measured in Fiji (Schindelin et al., 2012).

### 2.5 Picrosirius red staining and collagen birefringence quantification

Intact scWAT and pgWAT depots were excised from BL6 and HET3 mice, fixed in 10% buffered formalin overnight at room temperature and embedded in paraffin. Paraffin‐embedded tissues were sectioned 7 μm thick. Tissue sections were deparaffinized with HistoChoice Clearing Agent (Sigma‐Aldrich Cat#H2779) and hydrated in decreasing concentrations of EtOH (100%, 95% 70%, 30%, 0%). Tissues were stained in picrosirius red stain (Electron Microscopy Sciences Cat#26357) for 1 h, washed in two changes of acidified water (5 s each), dehydrated in three changes of 100% EtOH, cleared in histo‐grade Xylenes for 5 min and cover slipped using Permount mounting media (Electron Microscopy Sciences Cat#17986‐01). Five representative images per tissue were captured using both bright‐field and circularly polarized light to gauge collagen distribution. Total collagen was measured by first quantifying the area comprised by picrosirius red staining in the field of view using bright‐field microscopy. This was then divided by the area comprised exclusively of polarized collagen fibers. This provided a ratiometric value of collagen area that was unaffected by sample group variations in cell number and cell size which, as we have demonstrated here, vary greatly by sex and age. To differentiate the relative contribution of collagen fiber thicknesses, we adapted methods from Rich and Whittaker (2005) for use in Fiji (Schindelin et al., 2012). Hue values used for this analysis were as follows: green (52–128), yellow (39–51), orange (10–38), and red (0–9, 230–255). Saturation was not accounted for, and brightness was thresholded at 35–255 to prevent noise from being included in pixel counts. See Figure S3 for a schematic of this method.

### 2.6 Intraepidermal nerve fiber (IENF) immunostaining and quantification

Glabrous hind paw skin was excised and fixed in 2% Zamboni's fixative (Newcomer Supply # 1459A) for 2 h at room temperature, moved to 30% sucrose in 1XPBS overnight (until tissues sank), and embedded in OCT (Tissue‐Tek cat #4583 Miles, Inc.) with orientation noted. Tissues were sectioned at 25 μm onto glass slides. Tissue sections were blocked in 200 μl blocking solution (1× PBS/0.3% Triton X‐100/5% BSA) at room temperature for 1 h and then incubated in primary antibody (Proteintech rabbit anti‐PGP9.5 (14730‐1‐ap), 1:1000) for 1 h at room temperature and then moved to 4°C for incubation overnight. Sections were rinsed 3 × 1 h in 1XPBS and incubated with goat anti‐rabbit IgG Alexa Fluor Plus 594 (1:1000, ThermoFisher Cat#A32740) for 1 h at room temperature and then moved to 4°C for incubation overnight. Sections were rinsed with 3 × 1 h with 1× PBS and then incubated in 100 ng/ml DAPI solution for 15 min. Sections were rinsed 3 × 10 min in distilled H_2_O then mounted with a drop of mounting fluid (Prolong Gold, Thermofisher #P36931) and a No. 1.5 glass coverslip for imaging on a Leica Stellaris 5 confocal microscope. Region of interest (ROI) of the epidermis was generated for each image in Fiji (Schindelin et al., 2012). Fluorescence intensity was thresholded in Fiji using the intermodes function to remove skin autofluorescence from measurements. Nerve fiber density was measured as a ratio of PGP9.5 fluorescence to total ROI area for each tissue.

### 2.7 Neuromuscular junction (NMJ) immunostaining and quantification

Medial gastrocnemius (MG) and soleus (SOL) muscles were excised from male and female HET3 mice. Muscles were fixed in 2% PFA for 2 h, washed in 1× PBS, and muscle fibers were teased apart and placed between two glass slides held together by binder clips for 30 min at 4°C. Tissues were then blocked in 1× PBS/2.5% BSA/1% Triton X‐100 overnight at 4°C on rotator. The next day tissues were incubated in primary antibodies 2H3 (1:500, DSHB, Cat#2H3) and SV2 (1:250, DSHB, Cat#SV2) diluted in 1XPBS for 24 h at 4°C on rotator. The tissues were washed in 1XPBS for 4 h at 4°C with the 1XPBS replace hourly. Next, tissues were incubated in secondary antibody solution containing α‐Bungarotoxin (BTX) Alexa Fluor 555 conjugate (1:1000, ThermoFisher Cat#B35451) and goat anti‐mouse IgG1 Alexa Fluor 488 (1:500, ThermoFisher Cat#A‐21121) diluted in 1XPBS, incubated 24 h at 4°C on rotator. The next day tissues were washed in 1XPBS for 4 h with 1PBS replaced fresh every 1 h. When necessary, tissues were additionally stained with either SOX10 (1:250, Abcam Cat# ab227680), MPZ (1:250, Abcam, Cat#ab31851) or S100β (1:250, Abcam Cat#ab52642) for 24 h at 4°C on rotator, washed in 1XPBS, incubated in secondary antibody (goat anti‐rabbit IgG Alexa Fluor Plus 647, 1:500) for 24 h at 4°C on rotator, and then washed again in 1XPBS. Finally, tissues were placed onto a slide with three to free drops of aqueous mountant applied, sealed with coverslip, and imaged on a confocal microscope. Fifty NMJs were counted per tissue and were categorized as being fully occupied or not. The number of tSCs labeled by SOX10 was counted for each of those 50 NMJs when co‐staining was performed.

### 2.8 Microscopy

#### 2.8.1 Confocal

Confocal micrographs were captured on a Leica Stellaris 5, laser scanning confocal microscope using LASX software. Fluorescent labels were excited with either a diode 405 nm laser: DAPI (ex 405 nm, em 417–570) or a white light laser: Alexa Fluor 488 (ex 499 nm, em 510–570), Alexa Fluor 555 (ex 553 nm, em 565–610 nm), Alexa Fluor Plus 594 (ex 590 nm, em 600–700 nm), Alexa Fluor Plus 647 (ex 653 nm, em 665–720). Scanning speed was set to 400 Hz or 600 Hz. Photons were detected with Power HyD S detectors. Objectives included: HC PL APO 10×/0.40 CS2, HC PL APO 40×/1.30 OIL CS2, and HC PL APO 63×/1.40 OIL CS2. PinholeAiry 1.00 AU. Confocal zoom was applied to further increase magnification when necessary. Image processing performed in Leica Application Suite X and Fiji software.

#### 2.8.2 Widefield epifluorescence

Epifluorescence micrographs were captured on a Nikon Eclipse E400 epifluorescence microscope using a Hamamatsu ORCA‐Flash4.0 V2 Digital CMOS monochrome camera. Alexa Fluor 594 fluorophores were excited using a TxRed filter cube and Alexa Fluor 488 was excited with a GFP filter cube. Objectives used: Nikon CFI Plan Apo 10×/0.45 and Nikon CFI Plan Fluor 40×/0.75. Images were captured utilizing the extended depth of field (EDF) function; LUTs were adjusted to improve structural visualization. Post processing was performed in Nikon Elements BR software.

#### 2.8.3 Bright‐field and polarized

Bright‐field and polarized light micrographs were captured on a Nikon Eclipse E400 microscope using a Nikon DS‐fi3 color camera. Objectives used: Nikon CFI Plan Apo 4×/0.20, Nikon CFI Plan Apo 10×/0.45, Nikon CFI Plan Apo 20×/0.75, and Nikon CFI Plan Fluor 40×/0.75. LUTs were adjusted to improve structural visualization. Post processing was performed in Nikon Elements BR software.

### 2.9 Quantitative real‐time PCR

Zymo DirectZol RNA extraction kit (Zymo, Irvine, CA, USA; Cat#R2052) was used for RNA extraction from whole scWAT depot or similarly sized flank skin region. RNA yield was determined using a Nanodrop and cDNA was synthesized using High‐Capacity Synthesis Kit (Applied Biosystems, Foster City, CA, USA; Cat#4368813). Real‐time quantitative polymerase chain reaction was performed using SYBR Green (Bio‐Rad, Cat#1725271) on a CFX384 real‐time PCR detection system (Bio‐Rad, Hercules, CA, USA). Gene expression was normalized to housekeeper gene *Ppia* for analysis. Primers used for qPCR are listed in Table S1.

### 2.10 Whole adipose depot immunostaining, imaging, and quantification

Intact scWAT depots were excised from mice, fixed overnight in 2% PFA at 4°C, and processed following the Z‐depth reduction method as described previously (Willows et al., 2021) with accompanying protocol (Willows, Blaszkiewicz, & Townsend, 2022). Tissues were stained with the sympathetic nerve marker tyrosine hydroxylase (TH, 1:200, EMD Millipore, AB152), secondary antibody (goat anti‐rabbit IgG Alexa Fluor Plus 594, 1:1000, ThermoFisher Cat#A32740), and the vascular marker Isolectin IB_4_ conjugated to Alexa Fluor 488 (2.5 μg/ml, Thermofisher, Cat# I21411). Whole scWAT tissues were imaged on a Leica Stellaris 5 confocal microscope at 10× or 63× objective magnifications. Whole tissue images were generated by tiling Z‐maximum intensity projections of 10× objective magnification micrographs captured at 720 × 720 pixel resolution with a Z‐step size of 10 μm. When tiled together, whole depot image resolutions were roughly 30,000 × 19,000 pixels and were used for whole depot relative density quantifications and was reduced to an 800 pixel by linear binning (height × width ratio maintained) for visualization in figures. Tissue boundaries were outlined with the polygon sections tool in Fiji to create an ROI of the whole tissue which was used to measure the tissue area for normalization. TH fluorescence intensity was threshold at 35–255 and IB4 fluorescence intensity was threshold at 45–255 to generate masks for relative density measurements.

Fluorescence overlap was measured in five representative images of innervated blood vessels per tissue. Images were captured as 50 μm Z‐stacks (2.5 μm step size) at 10× objective magnification at 1024 × 1024 pixel resolution. A Gaussian blur (sigma = 80) was subtracted from each channel to remove background and a mask was generated for each channel separately. TH fluorescence intensity threshold was set to 100–255 to generate the mask for quantification. IB4 channel had an unsharp mask applied (radius = 1, mask = 0.60) and fluorescent intensity threshold was set to 15–255 to generate the first iteration of the mask. To further refine boundaries, a binary dilation was applied, a fill holes function was applied, and a median filter (radius = 1) was applied. Mander's overlap coefficient was measured between the two generated masks using the Coloc 2 plugin in Fiji and averaged for the five images per tissue.

### 2.11 Wire Myography

We utilized a Danish Myography Technologies A420 (Denmark) for wire myography experiments as previously described (del Campo & Ferrer, 2015). Mouse protocols for vascular analyses were approved by the Maine Medical Center Institutional Animal Care and Use Committee. Mice were heparinized with 300 U heparin injected intraperitoneally (Patterson Veterinary Cat#07‐893‐7851) 5 min prior to euthanasia. Mouse thoracic aortae with attached PVAT were excised in a 2‐mm segment and bathed in physiological saline solution (0.13 M NaCl, 4.7 mM KCl, 1.18 mM KH2PO4, 1.17 mM MgSO47H2O, 5.5 mM glucose, 0.026 mM EDTA, and 1.6 mM CaCl2) on ice. After mounting of with 40 μm wire, vessel segments were bathed in physiological saline solution under oxygenated conditions (95% O2 + 5% CO2) at 37°C for 30 min. Phenylephrine (Sigma Aldrich P6126) was used for vessel contraction at a dose curve from 2 nM to 10 μM. Every consecutive dose was added after vessel change reached a plateau. Vessels were washed twice for 15 min to restore basal tone, and pre‐contracted to 50%–80% of maximum phenylephrine‐induced contraction. Acetylcholine (Sigma‐Aldrich Cat#A2661) was used in a dose curve from 2 nM to 10 μM for relaxation. After acetylcholine‐induced vasodilation, vascular integrity was tested using 100 mM KCl, and peak contraction was recorded at 8 min post‐addition. EC50 calculations were performed using the non‐linear regression algorithm from GraphPad Prism v7.

### 2.12 Western blot (WB)

To confirm that the rapamycin treatment was effective at inhibiting mTORC1 in the late‐intervention group, we analyzed phosphorylation of p70 S6 kinase following methods described (Lamming et al., 2012). Protein expression was measured by Western blot analysis of liver lysates. Livers were homogenized in RIPA buffer with protease inhibitors in a Bullet Blender. A Bradford assay was performed to measure total protein from which equal concentrations of protein lysates were prepared in Laemmli buffer using 1× PBS as diluent. 30 μg of protein was loaded per lane of a 10% polyacrylamide gel, and following gel running, proteins were transferred to PVDF membrane and incubated with 10% Roche Blocking Reagent for 1 h at room temperature prior to antibody incubation. The membrane was bisected horizontally at 37 kDa to avoid the need for stripping proteins later. Primary antibodies included: p70 S6 Kinase (65 kda, 1:1000, Cell Signaling Cat#2708); p‐Thr389 p70 S6 Kinase (65 kda, 1:1000, Cell Signaling Cat#9234); Cyclophilin B (21 kDa, 1:40,000, Abcam, Cat#16045). Primary antibodies were incubated overnight at 4°C on a rotator with gentle agitation. Membranes were rinsed with 1× TBS‐T and then incubated in anti‐rabbit HRP‐linked secondary antibody (1:3000, Cell Signaling Technology, Cat#7074) for 1 h at room temperature. Blots were visualized with enhanced chemiluminescence (ECL; Pierce) on a Syngene G:BOX. Protein expression was normalized to Cyclophilin B and quantified by densitometry in Fiji (Schindelin et al., 2012).

### 2.13 Statistics

Statistical calculations for determining significance were made in Prism (GraphPad Software). ROUT outlier test (Q = 1%) was performed on raw data to identify and remove statistical outliers within datasets. Indicators of statistical significance were unpaired two‐tailed Student's *t*‐tests, Mann–Whitney nonparametric test, one‐ and two‐way ANOVAs with Tukey's correction for multiple comparisons, and Kruskal–Wallis nonparametric test with Dunn's correction for multiple comparisons (specified in each figure legend). Nonparametric tests were used for qPCR analysis of HET3 tissues due to a non‐Gaussian distribution of data points. Linear regression analysis was performed with goodness of fit measured by *R*‐squared and the significance of slope determined by *F*‐test. All error bars are SEMs. When *p*‐value is otherwise not directly stated: ^n.s.^ *p* > 0.05, *p < 0.05, **p < 0.01, ***p < 0.001, ****p < 0.0001.

## 3 RESULTS

### 3.1 Age‐induced changes to adiposity, adipocyte cell size, and adipose tissue inflammation

Aging precipitates several phenotypic changes to adipose tissue in both mice and humans, including adipose tissue redistribution (subcutaneous depots shrink while visceral depots expand), chronic inflammation, and reduced WAT browning potential, for example (Mancuso & Bouchard, 2019; Palmer & Kirkland, 2016; Von Bank, Kirsh, & Simcox, 2021). In mice, the majority of these studies were performed using genetically inbred strains such as BL6. Our goal was to investigate these age‐related changes in genetically diverse HET3 mice that are used for systematic longevity treatment studies, and serve as a more appropriate mouse model for human aging. Additionally, we also collected data in the well‐studied BL6 mouse as an inbred‐strain reference. Male mice are more prone to weight gain, macrophage infiltration and insulin resistance when placed on a high‐fat diet (Chang et al., 2018). Additionally, male rats show an increase in age‐related metabolic disturbances, such as insulin resistance and WAT inflammation compared females (Garcia‐Carrizo et al., 2017). Taken together male WAT is less accommodating to energy imbalance and takes on pathophysiological states earlier than in females. For these reasons, we focused our assessment of aging in BL6 male mice, as a reference for HET3 sex difference assessments.

**FIGURE 1:**
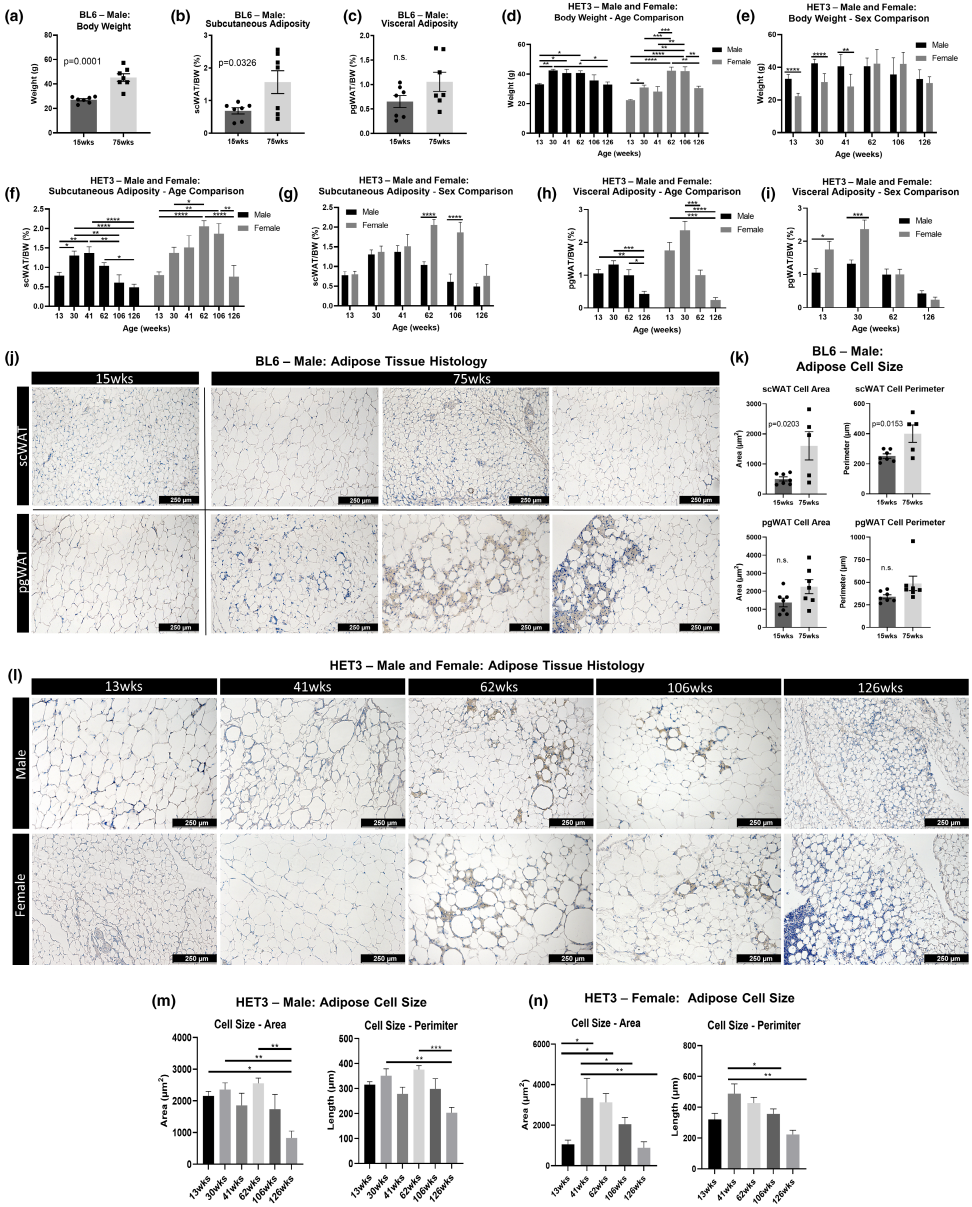
Genetic background influences on adiposity with aging. Male C57BL/6J (BL6) mice at 15 and 75 weeks of age were compared for body weight (a). Subcutaneous white adipose tissue (scWAT) and perigondal (pg)WAT weights were used to calculate subcutaneous adiposity (scWAT/body weight) (b) and visceral adiposities (pgWAT/body weight) (c). Male and female HET3 mice at 13, 30, 41, 62, 106, and 126 weeks were compared by age or sex for body weight (d,e), subcutaneous adiposity (f,g), and visceral adiposity (h,i). Hematoxylin staining was performed on BL6 mouse axillary (ax)‐scWAT and pgWAT (j) and cell sizes were quantified by area and perimeter (k). HET3 pgWAT was stained with hematoxylin (l) and cell size was quantified for males (m) and females (n). Three representative images were captured per tissue, quantified, and averaged per tissue per animal (j–n). BL6 mice; *N* = 3–7. HET3 mice; *N* = 5–12. Unpaired two‐tailed Student's *t*‐test (a–c,k). Two‐way ANOVA with Tukey's correction for multiple comparisons (d–i). One‐way ANOVA with Tukey's correction for multiple comparisons (m,n). Error bars are SEMs. ^n.s.^ *p* > 0.05, *p < 0.05, **p < 0.01, ***p < 0.001, ****p < 0.0001.

Male BL6 mice at 15 weeks and 75 weeks (*N* = 7 at each age) were measured for body weight (Figure 1a), subcutaneous adiposity (scWAT weight/body weight; Figure 1b), and visceral adiposity (pgWAT weight/body weight; Figure 1c). Adipose depot weights used for calculating adiposity can be found in Figure S1. Body weight was increased significantly at 75 weeks of age versus 15 weeks (*p* = 0.0001), which more closely correlated with an increase in subcutaneous adiposity (*p* = 0.0326) and not visceral adiposity (Figure 1b,c).

Male and female HET3 mice were aged to 13, 30, 41, 62, 106, and 126 weeks (*N* = 5–12 per group depending on cohort survival). Body weights and adiposities were measured at these ages and compared by age and sex. Male and female HET3 mice both displayed a significant increase in body weight from 13 to 30 weeks (male, *p* = 0.0022; female, *p* = 0.0128; Figure 1d). After 30 weeks, males steadily lost body weight, whereas females had a secondary increase in body weight between 41 and 62 weeks (*p* = 0.0008). However, comparing males to females at 62 weeks, the body weights were the same, but at 13 and 30 weeks of age, male mice weighed more than females (Figure 1e). Therefore, the 41‐ to 62‐week weight gain in females served to blunt the initial weight difference between the sexes.

Changes in HET3 scWAT adiposity with age mimicked the trends observed in body weight for both sexes (Figure 1f), with females displaying a much greater proportion of scWAT than males at 62 weeks (*p* < 0.0001) and 106 weeks (*p* < 0.0001; Figure 1g). By contrast, visceral adiposity continuously decreased with age in both sexes (Figure 1h), and females had higher visceral adiposity than males at 13 weeks (*p* = 0.0183) and 30 weeks (*p* = 0.0002; Figure 1i). Taken together, the genetically diverse HET3 mouse line displayed a striking sexual dimorphism in age‐related body weight and adiposity trajectories that may cause, or reflect, underlying changes to health outcomes.

In females, irregular hormone cycling signals the start of reproductive senescence which occurs at approximately 8 months (32 weeks of age). In the majority (70%) of mice, this is followed by a polyfollicular anovulatory state of constant estrus characterized by sustained levels of plasma 17β‐estradiol and low levels of progesterone, which is followed by anestrous several weeks later, which is characterized by low circulating ovarian hormones (Diaz Brinton, 2012). It is possible that the increased subcutaneous adiposity observed in females is a result of the increased circulating hormones at those ages, as estrogens have been linked to the promotion, maintenance, and control of adipose tissue metabolism (Pallottini et al., 2008).

Hematoxylin staining of pgWAT and scWAT sections from BL6 male mice demonstrated several age‐related changes (Figure 1j). Cell size appeared visually smaller in both depots at 75 weeks, but was only statistically significant for scWAT (area, *p* = 0.0203; perimeter, *p* = 0.0153; Figure 1k). Most importantly, pgWAT and scWAT appeared visually distinct from one another at 75 weeks, as scWAT lacked the increase in crown‐like structures and lipofuscin that were observed in pgWAT at this age. These provide supporting evidence that aging pathologies can vary between adipose depots (Blaszkiewicz et al., 2020).

HET3 pgWAT tissue sections looked similar to those of BL6, with numerous crown‐like structures and patches of lipofuscin appearing at 62 weeks of age and later (Figure 1l). In general, cell size followed the patterns displayed by changes in adiposity (Figure 1m); however, several hypertrophic adipocytes were dispersed throughout the tissue and were surrounded by patches of lipofuscin and crown‐like structures. This was observed primarily in females (Figure 1l, female at 106 weeks).

### 3.2 Aging impacts peripheral nerves in skin and muscle

Aging is associated with increased rates of peripheral neuropathy in both mice (Yezierski, 2012) and in humans (Richardson, 2002), including in adipose tissue (Blaszkiewicz et al., 2019). Studies in mice have been limited to investigating neuropathy in inbred‐strains and have not yet been undertaken in genetically diverse HET3 mice, despite the likelihood that genetic background may contribute to age‐related tissue neuropathy.

**FIGURE 2:**
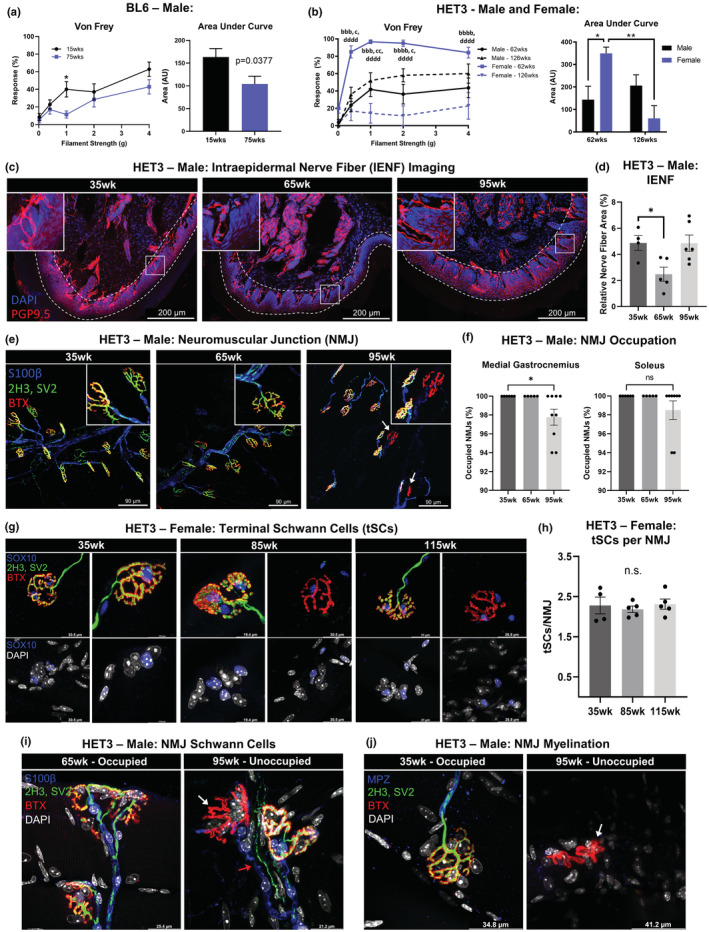
Age‐related neuropathy. Von Frey tactile allodynia test was performed on male BL6 mice at 15 and 75 weeks (a) and both male and female HET3 mice at 62 and 126 weeks (b). Compared by age and/or sex at each filament strength as well as for the area under each curve (a,b). Male HET3 mice at 35, 65, and 95 weeks had hind paw skin assessed for intraepidermal nerve fiber (IENF) density via immunolabeling and confocal imaging. Peripheral nerves (PGP9.5) and nuclei (DAPI) (c). Epidermal cell layer indicated by dashed lines (c). Relative nerve fiber density was quantified as the area of TH labeling normalized to epidermal area (d). Medial gastrocnemius and soleus muscles were stained for neuromuscular junction (NMJ) occupation nerve/pre‐synapse (SV2, 2H3), post‐synapse (BTX), and myelin (MPZ) (e). NMJ occupation was quantified and compared by age; 50 NMJs were counted per tissue (f). Terminal Schwann cell (tSC) nuclei were labeled (SOX10) in female HET3 mice at 35 weeks, 85, and 115 weeks and displayed tSCs located at occupied, altered, and unoccupied NMJs (g). Average number of tSCs per NMJ for 50 NMJs (h). NMJs were co‐stained with markers for Schwann cells (S100β) (i) and myelination (MPZ) (j). White arrows point no unoccupied NMJs and red arrow points to Schwann cell leading to unoccupied NMJ (i,j). BL6 mice; *N* = 7. HET3 mice; *N* = 4–12. Unpaired two‐tailed Student's *t*‐test (a). Two‐way ANOVA with Tukey's correction for multiple comparisons (b). One‐way ANOVA with Tukey's correction for multiple comparisons (d,f,h). Error bars are SEMs. ^n.s.^ *p* > 0.05, *p < 0.05, **p < 0.01, ***p < 0.001, ****p < 0.0001. a = male‐62 weeks:male‐126 weeks; b = female‐62 weeks:female‐126 weeks; c = male‐62 weeks:female‐62 weeks; d = male‐126 weeks:female‐126 weeks

Since small fiber peripheral neuropathy in skin and subdermal tissues, the type of neuropathy most associated with aging, is thought to begin with the longest axons that innervate the skin of the extremities, we assessed paw skin sensory nerve function through the standard von Frey tactile sensitivity and nociception test, a classical measure for sensory peripheral neuropathy. This revealed that BL6 males had reduced sensitivity as they aged from 15 to 75 weeks (Figure 2a). Interestingly, a focused assessment at the branch point of sexually dimorphic phenotypes in weight and adiposity (62 weeks) showed that female HET3 mice were protected from neuropathy at middle age (62 weeks; Figure 2b), while at later age (126 weeks), they fared worse than the males. This further supports the observation that females have delayed onset of aging‐related morbidities, which are then exacerbated compared to males at late age, and are likely linked to hormonal changes after reproductive senescence.

This behavioral nerve testing was supported by histological staining of skin intraepidermal nerve fibers (IENF) using the pan‐neuronal marker PGP9.5 (Figure 2c, area within dashed line) and was quantified as relative nerve fiber area (Figure 2d). Male HET3 mice had decreased IENF from 35 to 65 weeks of age (*p* = 0.0358; Figure 2d). Compared to the hind paw skin of 35‐week‐old mice, in 65‐week‐old mice, the dermal fibers were still evident within the subepidermal neural plexus (Figure 2c), but a paucity of fine fibers branching into the epidermal layer was noted. However, at late age (95 weeks old), IENF density increased back to baseline. This pattern of paw skin innervation looks strikingly similar to what was previously observed in BL6 mice (Verdu et al., 2000). We suspect that the increase in IENF density could be due to a pathologic aberrant outgrowth of small fibers in late‐stage peripheral neuropathy (Hirai et al., 2017). This would at least partially explain why we observe an increase in paw innervation in old age, but not an increase in the response to mechanical stimuli.

Finally, neuromuscular junction (NMJ) integrity was assessed by histology and quantification of occupied versus unoccupied junctions in male HET3 mice (Figure 2e). Staining revealed significant NMJ neuropathy in the medial gastrocnemius (MG) muscle (*p* = 0.0486), but not the soleus (SOL) muscle (Figure 2f). Similar MG neuropathy was observed in female HET3 mice (*p* = 0.0151) (Figure S2a). Decreased NMJ occupation by 95 weeks was comparable to what had been shown in BL6 mice previously (Valdez et al., 2010). Co‐staining NMJs with the Schwann cell nuclear marker SOX10 was performed to see if the age‐related NMJ neuropathy in HET3 mice could be attributed to a loss of terminal Schwann cells (tSCs, or specialized support cells located at the NMJ terminus) as had been proposed previously (Fuertes‐Alvarez & Izeta, 2021). At 85 and 115 weeks of age, we noted an increase in structurally altered and unoccupied NMJs (Figure 2g), as anticipated (Taetzsch & Valdez, 2018). However, tSCs were located at all NMJs regardless of NMJ morphology or pre‐synaptic occupation (Figure 2g), and the number of tSCs per NMJ was unchanged among all ages (Figure 2h). We additionally performed co‐labeling of NMJs with the Schwann cell membrane marker S100β or the myelin marker MPZ to parse out potential differences with age. Interestingly, the Schwann cells continued to form a path to NMJ's, even when the nerve ending itself was absent in aged animals (Figure 2i, red arrow). This sustained Schwann cell presence appeared to have no impact on de‐myelination, as myelin was absent around unoccupied NMJs (Figure 2j).

### 3.3 Changes in adipose tissue and skin gene expression vary by age and sex

We previously reported adipose tissue neuropathy with aging in mouse and human samples (Blaszkiewicz et al., 2019). Now, in order to investigate potential contributions of tissue gene expression changes across aging in the HET3 mouse as factors contributing to age‐related adipose neuropathy, we developed a panel of qPCR markers to investigate cytokines (interleukin 4, *Il4*; interleukin 6, *Il6*; interleukin 10, *Il10*; interleukin 13, *Il13*), angiogenesis markers (platelet endothelial cell adhesion molecule, *Pecam1/Cd31*; vascular endothelial growth factor a, Vegfa), Schwann cells (*Sox10*), nerve terminals (post‐synaptic density 95, *Psd95*; glutamate ionotropic receptor AMPA type subunit 2, Gria2; Synapsin I, *Syn1*; Synapsin II, *Syn2*; Synaptophysin, *Syp*), mitochondrial respiration (ATP synthase, *Atp1*; Ubiquinol‐cytochrome c reductase, *Uqcr*; cytochrome c oxidoreductase Vb, *Cox5b*; NADH dehydrogenase, *Ndufa1*), and collagens (collagen type 1 α1, *Col1a1*; collagen type 2 α1, *Col2a1*; collagen type 3 α1, *Col3a1*; collagen type4 α1, *Col4a1*; collagen type 5 α1, *Col5a1*; collagen type 6 α1, *Col6a1*).

**FIGURE 3:**
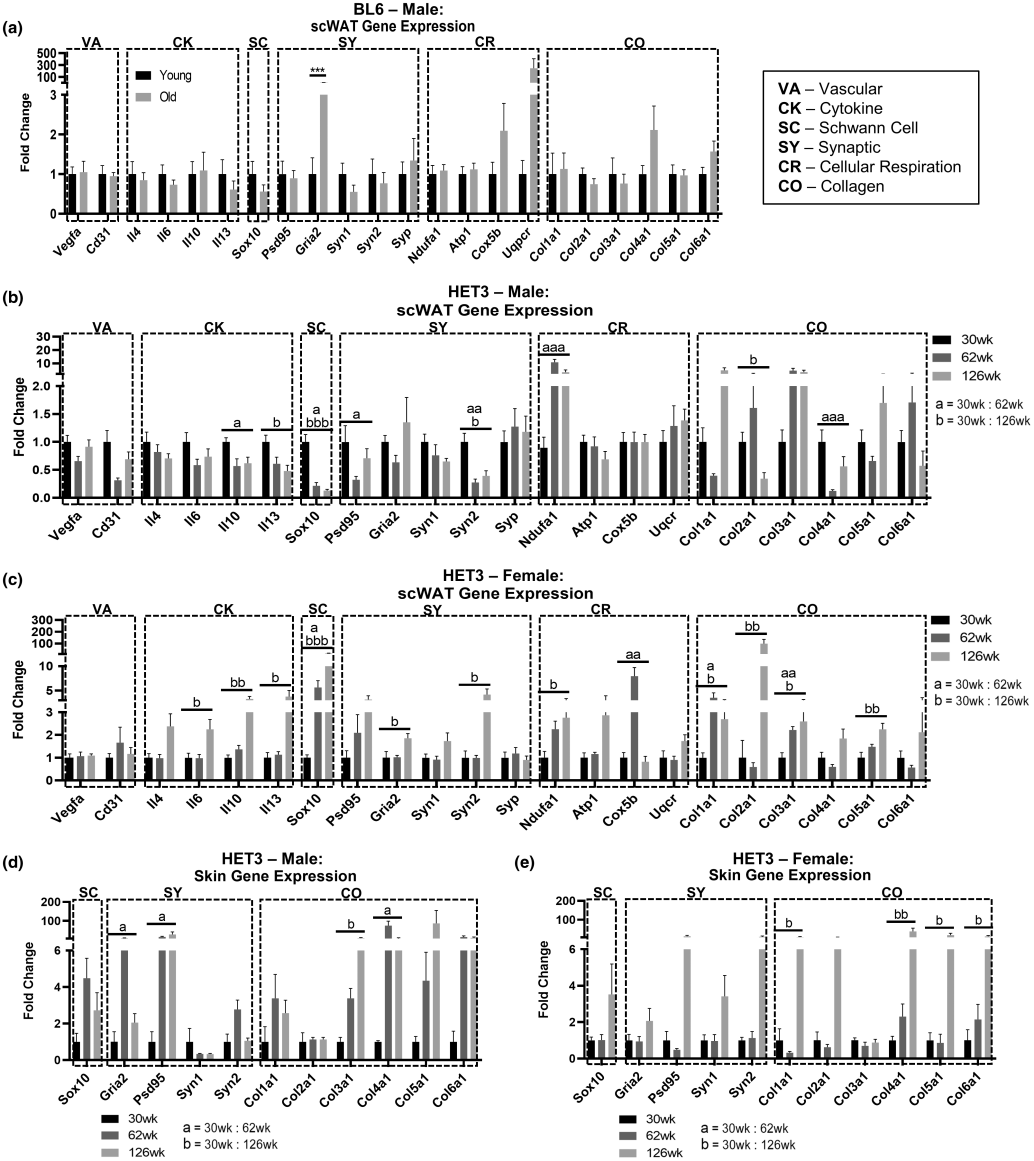
Adipose tissue and skin gene expression changes across aging. Gene expression by qPCR in axillary scWAT from male BL6 mice (a). Gene expression in axillary scWAT of HET3, males (b) and females (c). Gene expression in axillary scWAT of HET3 in males (b) and females (c). Gene expression of HET3 flank skin in males (d) and females (e). Genes organized into functionally similar groups: vasculature (VA), cytokines (CK), Schwann cell (SC), synaptic (SY), cellular respiration (CR), and collagen (CO). BL6 mice; *N* = 6–7. HET3 mice; *N* = 5–7. Unpaired two‐tailed Student's *t*‐test (a). Kruskal–Wallis nonparametric test with Dunn's post hoc for multiple comparisons, corrected *p*‐values reported (b–d). Error bars are SEMs. ^n.s.^ *p* > 0.05, *p < 0.05, **p < 0.01, ***p < 0.001, ****p < 0.0001. a = 30 weeks:60 weeks, b = 30 weeks:126 weeks

We measured gene expression in whole scWAT tissue lysates from BL6 (15 weeks; 75 weeks; Figure 3a) and HET3 (30 weeks; 62 weeks; 126 weeks; Figure 3b,c) mice. Interestingly, male BL6 mice only displayed a change in *Gria2* expression, which was increased at 75 weeks (*p* = 0.0002). Male HET3 mice displayed a marked decrease in *Sox10* and *Syn2* (with *Syn1* trending down) with advancing age, which may indicate adipose neuropathy. Male HET3 mice displayed a coordinated decreasing trend for all cytokine markers with increasing age, with significant decreases in *Il10* and *Il13* (Figure 3b), together suggesting age‐induced tissue inflammation. The variation in expression across individuals may be due to the mixed genetic background of this line, and the impacts of genetics on gene expression (Lipman et al., 2004). Results from female HET3 scWAT gene expression were opposite of the male mice (Figure 3c), again indicative of protection from age‐related pathologies. Cytokine gene expression revealed a coordinated increase for *Il4*, *Il6*, *Il10,* and *Il13* at 126 weeks of age, with all but *Il4* being statistically significant. In females, there was also a marked increase in *Sox10* and *Syn2* (with *Syn1* trending up), indicating compensatory support for tissue innervation. Like BL6 mice, female HET3 mice had increased *Gria2* expression, and if this represents loss of tissue nerve endings, the upregulation of Schwann cell and other synaptic markers may indicate enhanced neural plasticity in females to counteract this onset of age‐related neuropathy. Mitochondrial markers *Ndufa1* and *Cox5b* increased with age in females, and collagen gene expression increased for all collagen types by 126 weeks, with *Col1a1*, *Col2a1*, *Col3a1*, and *Col5a1* all reaching statistical significance (Figure 3c).

Additionally, in HET3 mice, we measured gene expression of many of these same markers in the “flank” skin directly above the inguinal scWAT depot (Figure 3d,e), since age‐related small fiber peripheral neuropathy likely starts at the skin surface (length‐dependent neuropathy of the longest axons first) and then progresses down to deeper tissue layers over time. Male mice displayed increased *Psd95* expression, which may represent peripheral neuropathy in the tissue since shedding of PSD95 from nerve terminals can occur with loss of tissue innervation and synaptic remodeling (Low & Cheng, 2006). Also observed was an increase in *Gria2* expression with age and a trend for increased collagen expression overall, with just *Col3a1* and *Col4a1* being significant (Figure 3d). Similar to scWAT, female HET3 skin displayed consistent increasing trends for synaptic, Schwann cell, and collagen gene expression at 126 weeks of age, but none of these changes were significant (Figure 3e).

The dissimilarities between male and female HET3 gene expression in scWAT and the overlying skin provided further evidence for sexual dimorphism in aging. Females seem to be protected from mid‐age peripheral neuropathy, and increased Schwann cell and synaptic gene expression may be the contributing (or resulting) factors. While direct comparisons cannot be made between BL6 and HET3 mice, the stark contrast in gene expression patterns further emphasized the impact that genetic variability, or lack there‐of, may have on age‐related pathologies, including neuropathy.

### 3.4 White adipose tissue becomes fibrotic with age regardless of genetic background

Fibrosis is defined as the excess accumulation of extracellular matrix (ECM) components, primarily collagens type I, III, and VI (Sun et al., 2013). In fibrotic states in adipose tissues, collagen limits adipocyte cell growth causing constriction and reducing the morphological flexibility that is characteristic of adipose tissue cells. Fibrosis in adipose is especially prevalent with obesity (Khan et al., 2009). This mechanical constriction due to increased ECM can lead to cell damage and chronic inflammation, and adipose tissue fibrosis has been linked to insulin resistance, chronic inflammation, and impaired adipogenesis (Datta et al., 2018; DeBari & Abbott, 2020; Khan et al., 2009). Much focus has been placed on investigating adipose fibrosis with obesity due high‐fat/high‐sugar diets (Pincu et al., 2016), but little work has been done with aged adipose, despite the fact that aging has been demonstrated to increase the likelihood of pathological cardiac and pulmonary fibrosis in mice and humans (Murtha et al., 2019). Infrequent studies to date have reported increased fibrosis in visceral and dermal WAT with aging (Donato et al., 2014).

**FIGURE 4:**
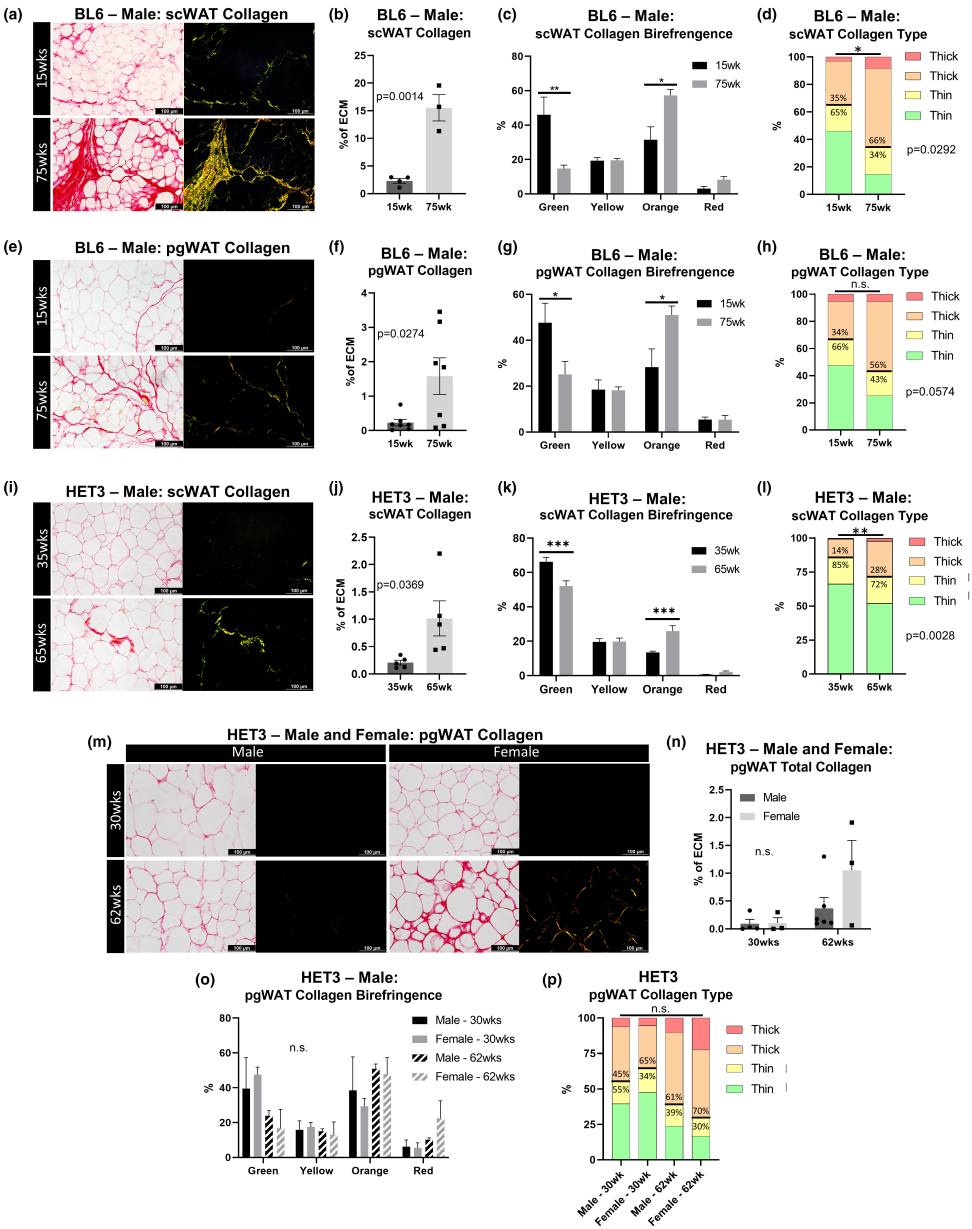
Adipose tissue collagen distribution across aging. Picrosirius red (PSR) collagen staining and quantification of 7 μm thick adipose tissue sections. Male BL6 scWAT (a–d). Male BL6 pgWAT (e–h). Male HET3 scWAT (i–l). Male and female HET3 pgWAT (m–p). Representative images of the same regional tissue area were captured separately with bright‐field and polarized light at 20× objective magnification (a,e,i,m). Five representative images were captured per tissue per animal. Total collagen was measured as a ratio of birefringent collagen to total PSR staining (b,f,j,n). Changes in specific hues of collagen birefringence (c,g,k,o). Contribution of collagen fiber thickness determined by hue (d,h,l,p). Thin collagen fibers (green and yellow); thick collagen fibers (orange and yellow). BL6 mice; *N* = 3–7. HET3 mice; *N* = 3–6. Unpaired two‐tailed Student's *t*‐test (b,d,f,h,j,l). Two‐way ANOVA with Tukey's correction for multiple comparisons (c,g,k,n,o). One‐way ANOVA with Tukey's correction for multiple comparisons (P). All error bars are SEMs. ^n.s.^ *p* > 0.05, *p < 0.05, **p < 0.01, ***p < 0.001, ****p < 0.0001

We investigated changes to collagen deposition in both BL6 and HET3 mice in visceral and subcutaneous adipose depots with age. To do so, we developed a method for quantifying total collagen and the ratio of thin to thick collagen fibers in adipose tissue, that is not confounded by the sex‐ and age‐specific changes in adipocyte cell size and cell number (Figure S2). We used picrosirius red (PSR) staining, which binds to type I, II, and III collagens (Rittie, 2017), coupled with polarized light microscopy to differentiate collagen fiber thickness in the tissue ECM (Lattouf et al., 2014). A striking increase in total collagen was observed in BL6 mouse scWAT with increasing age (Figure 4a,b, *p* = 0.0014). The color of collagen birefringence indicated the thickness of collagen fibers, with green and yellow representing thin fibers and orange and red representing thick fibers (Rich & Whittaker, 2005). Although collagen fiber thickness has been historically used to distinguish between type I (thick) and type III (thin) collagens (Junqueira et al., 1978), there are several instances when this is not a direct correlation (Rich & Whittaker, 2005), so we referred to fibers as “thick” or “thin” in our analyses. With increased age, green birefringence decreased (*p* = 0.0121), while orange birefringence increased (*p* = 0.0108; Figure 4c), and the ratio of thin to thick collagen (15 week, 65%:35%) decreased significantly with age (75 week, 34%:66%; *p* = 0.0292; Figure 4d). BL6 pgWAT demonstrated identical changes with age, but less collagen was observed overall (Figure 4e–h).

Heterogeneous mouse strain (HET3) mice appeared to have less scWAT collagen than BL6 mice (Figure 4i), likely a protective effect of their diverse genetic background, but aging from 35 weeks to 65 weeks increased total collagen in the tissue (*p* = 0.0369; Figure 4j). The proportion of green birefringence decreased, and orange increased with age (green, *p* = 0.0001; orange, *p* = 0.0007; Figure 4k). Accordingly, the ratio of thin to thick collagen (35 week, 85%:14%) decreased significantly with age (66 weeks, 72%:28%; *p* = 0.0028; Figure 4l). In addition to comparing HET3 pgWAT collagen across age, we also compared males to females. Although the females at 62 weeks appeared to become fibrotic (Figure 4m), this difference was not statistically significant in either sex (Figure 4n). Trends in collagen birefringence and ratios of thin to thick collagen also lacked statistical significance (Figure 4o,p). These data confirm that both subcutaneous and visceral adipose depots become fibrotic with age, regardless of the mouse strain, sex, and that scWAT is more affected than pgWAT.

### 3.5 Aging impacts on vasculature

**FIGURE 5:**
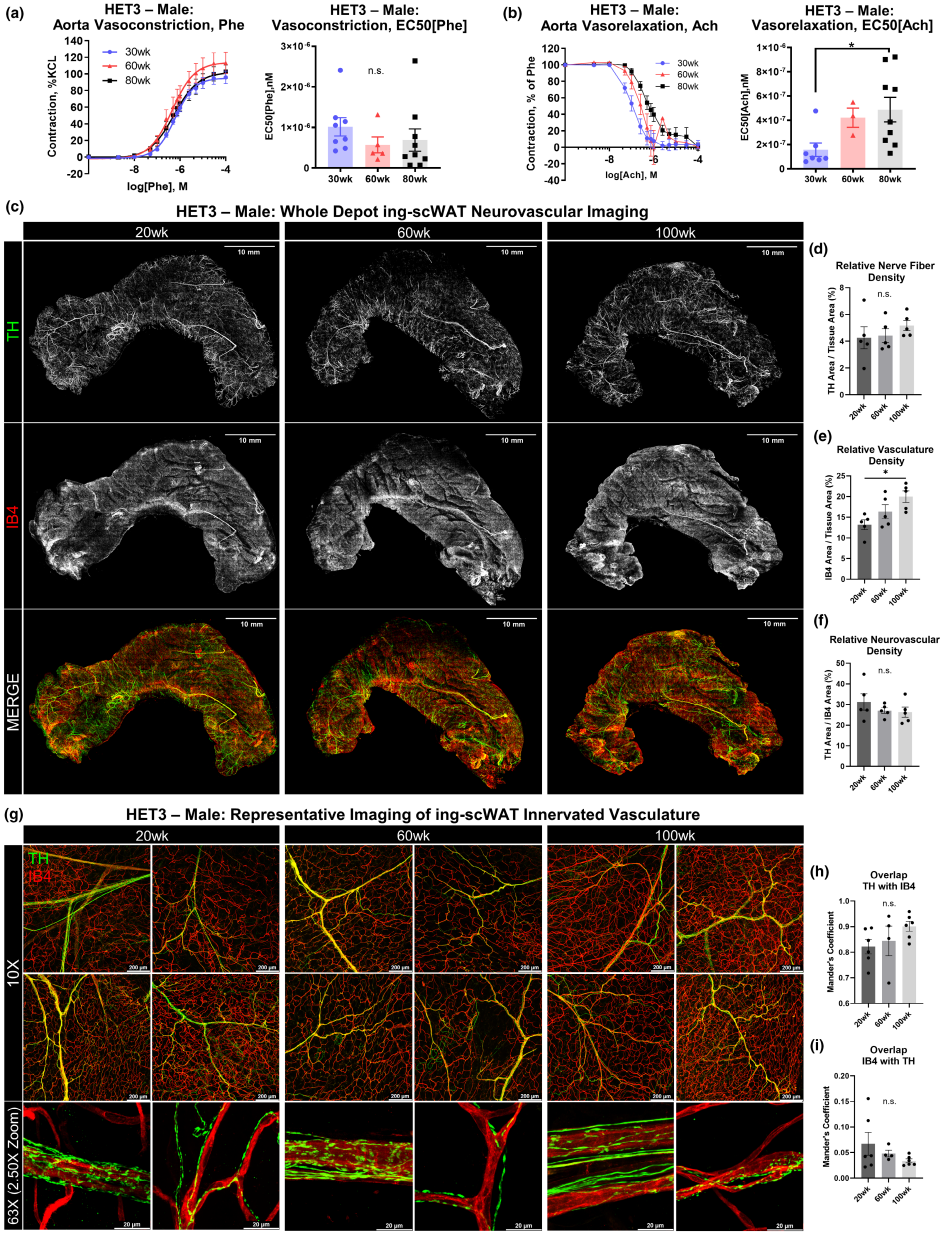
Vascular changes with aging. Wire myography of male HET3 aortic vasoconstriction and (a) and vasorelaxation (b) at 30, 60, and 80 weeks (*N* = 3–9). Segments of thoracic aorta were contracted with a dose response of phenylephrine. Vessels were then returned to basal tone, and pre‐contracted to 50%–80% maximal phenylephrine‐induced contraction. Dose response curve of acetylcholine was performed to measure vasorelaxation (a). Vasocontraction was normalized to maximal KCl contraction, and vasorelaxation was calculated as percentage of Pre‐contraction (B). EC50 for contraction and relaxation were calculated (a,b). Intact inguinal scWAT depots were excised from male HET3 mice at 20, 60, and 100 weeks and labeled for nerves (tyrosine hydroxylase, TH) and blood vessels (isolectin IB_4_, IB4). Micrographs of whole tissues were generated from 10× objective magnification confocal images that were tiled together and Z‐maximum intensity projected (c). Representative images of *N* = 5 tissues are displayed (c). Relative nerve fiber density for the whole tissue was calculated as TH‐labeled area normalized to total tissue area (d). Relative vascular density for the whole tissue was calculated as IB4‐labeled area normalized to total tissue area (e). Relative neurovascular area was calculated as TH area normalized to IB4 area (f). Higher magnification representative maximum intensity projection images were captured at 10× objective magnification or 63× objective magnification with an additional 2.50× confocal zoom applied (g). Colocalization of nerve–blood vessel overlap was performed by comparing Mander's coefficients between groups. Higher values correspond to greater overlap. Overlap was calculated from the 10× objective magnification images of the intact whole tissues (*N* = 4–6, *n* = 5) (h,i). Scale bars are 10 mm (c), 200 μm and 20 μm (g). One‐way ANOVA with Tukey's correction for multiple comparisons. All error bars are SEMs. ^n.s.^ *p* > 0.05, *p < 0.05, **p < 0.01, ***p < 0.001, ****p < 0.0001

Based on our previously reported observations of peripheral nerves around blood vessels in scWAT reducing with age in male BL6 mice (Blaszkiewicz et al., 2019), we wanted to determine whether this was also observed in a more genetically heterogeneous mouse strain (HET3). We first performed wire myography of thoracic aortae from male HET3 mice at approximately 30 weeks, 60 weeks, or >80 weeks of age. Because of the impact of the surrounding perivascular adipose tissue on vascular reactivity, these assays were performed on isolated aortic segments with overlying perivascular adipose intact. No difference between ages were observed in total vasoconstriction when induced with phenylephrine (Phe; Figure 5a). Vasorelaxation was induced with acetylcholine (Ach) following constriction with Phe, and this did reveal a steady decrease in relaxation potential with age that became significantly blunted at 80 weeks (*p* = 0.0189; Figure 5b). This was similar to what had been observed in aged rats (Luttrell et al., 2020), and similar to a previous study which demonstrated that B6D2F1 mice (which comprise 50% of the HET3 genome) displayed decreased insulin‐stimulated arterial vasodilation in scWAT, pgWAT, and BAT with age (Islam et al., 2020). Perivascular adipose tissue phenotype was assessed across aging, using a previously established reproducible protocol to quantify percentage lipid within mouse PVAT (Tero et al., 2022). Interestingly, there were no differences in percent lipid in PVAT between mouse ages, but lower lipid in PVAT was associated with body weight (Figure S3a,b).

To follow up on our prior observation of nerves dying‐back from ing‐scWAT vasculature in aged male BL6 mice we investigated the occurrence of neurovascular neuropathy in the HET3 mouse through quantitative histology. We excised intact whole inguinal scWAT depots from male HET3 mice (20, 60, and 100 weeks old) and co‐stained for tyrosine hydroxylase (TH, the rate‐limiting step in catecholamine synthesis) to label sympathetic nerves and Isolectin‐Ib_4_ (IB4) to label blood vessels (a caveat being that IB4 may also label a small subset of non‐peptidergic nociceptive neurons, which are easily distinguishable from the vasculature by morphology). Fluorescence area of Z‐maximum intensity projections of the whole tissue was measured for nerve and blood vessels. We found that tissue innervation and vascularity somewhat increased with increased tissue mass (Figure S4c), contrary to what was observed in the obese, diabetic, and neuropathic BTBR *ob/ob* mouse model (Wang et al., 2020; Willows, Gunsch, et al., 2022). Potentially, a relatively slow increase in fat mass (as with aging) allows the nerves and vasculature to respond adequately to tissue demands, in contrast to obese states. To account for this slight increase, total innervation and vascularity were normalized to total tissue area, which eliminated any correlation between fluorescence area and tissue mass (Figure S4c). We found that age had no impact on total nerve fiber density as averaged across the whole tissue (although regional patterns were observed to be different across age; Figure 5d), but that tissue vascularity did increase by 100 weeks of age (Figure 5e). Since the relative innervation of an ing‐scWAT depot correlated to the relative vascularity (Figure S4d), the disconnect between increasing vascularity and unchanged innervation could be reflected in a significantly altered ratio of nerves to blood vessels at 100 weeks. However, this was not the case (Figure 5f). To investigate further, we analyzed tissue vasculature at higher magnifications to look for signs of neuropathy, such as nerves disassociated from blood vessels, as previously observed in BL6 mice (Blaszkiewicz et al., 2019). No such signs were observed (Figure 5g) in the HET3 mice, again potentially a protective effect of the mixed genetic background. Quantification of nerve–blood vessel overlap further supported that there was no change in blood vessel innervation with age (Figure 5h,i). While extensive quantification was only performed in males, histology of female HET3 mice at 62 and 126 weeks also did not reveal any signs of adipose tissue neuropathy (Figure S4e). Likely, inter‐individual differences due to relative genetic contributions of the four founder strains contributed to the blunting of any neuropathic phenotype in adipose compared to BL6 mice, which may be a strain more prone to peripheral neuropathy with aging.

### 3.6 Aging impacts on the neuro‐adipose nexus (NAN)

**FIGURE 6:**
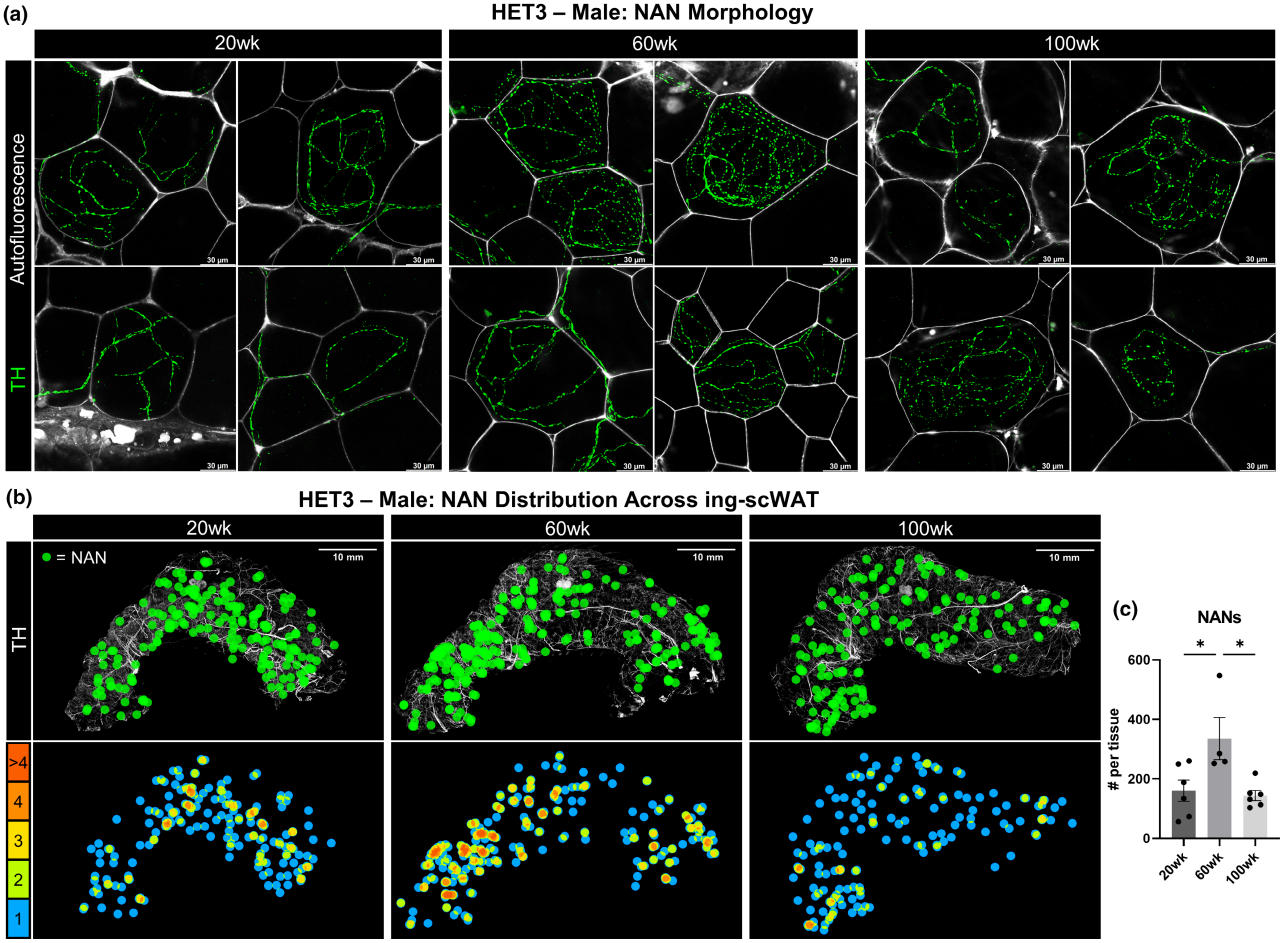
Neuro‐adipose nexus (NAN) distribution fluctuates with age. Intact inguinal scWAT depots were excised from male HET3 mice at 20, 60, and 100 weeks and labeled for nerves with TH. NANs were identified by densely varicose axons innervating single adipocytes (visualized with autofluorescence). NAN morphology compared across ages (a). NAN distribution across whole ing‐scWAT depots displayed as a representative tissue from each age group. Individual NANs labeled by a green dots superimposed over the intact tissue (b). Adjacent dot overlap was color coded and displayed below (b). Total number of NANs was counted for each tissue (*N* = 4–6) (c). Scale bars are 30 μm (a) and 10 mm (b). One‐way ANOVA with Tukey's correction for multiple comparisons. All error bars are SEMs. ^n.s.^ *p* > 0.05, *p < 0.05, **p < 0.01, ***p < 0.001, ****p < 0.0001

With our recent discovery of specialized synaptic vesicle‐containing nerve terminals in ing‐scWAT, the neuro‐adipose nexus (NAN) (Willows et al., 2021; Willows, Gunsch, et al., 2022), we concluded our analysis of adipose tissue innervation by assessing changes in NAN structure and/or number with age in 20‐, 60‐, and 100‐week‐old male HET3 mice. NANs were observed in all ing‐scWAT depots regardless of age; visualized by varicose axons clustering around specific adipocytes (visualized by their autofluorescence; Figure 6a). There was a striking increase in NANs with age, ranging from an average of ~160 per tissue at 20 weeks to ~335 per tissue at 60 weeks old (*p* = 0.0260). By 100 weeks, the number of NANs per tissue returned to baseline at ~144 (*p* = 0.0157; Figure 6b). The twofold increase in NANs at middle age was not reflected by nerve fiber density changes (Figure 5), and moreover, we found that the number of NANs per tissue did not correlate to ing‐scWAT weight (Figure S5a), relative nerve fiber density (Figure S5b), nor relative vascular density (Figure S5c). This provided strong evidence for the coordinated redistribution of tissue innervation in response to changing metabolic needs associated with aging and may underscore that nerve remodeling aberrations and not just loss of total neurite density in the tissue, may indicate a pathologic state in adipose. However, this serves as the first piece of data showcasing the neuroplastic potential of NANs.

### 3.7 Longevity treatment rapamycin triggered significant body weight loss in males with loss in adiposity only in females

To investigate the effects of the anti‐aging treatment rapamycin on adipose tissue and age‐related peripheral neuropathy, male and female HET3 mice were fed rapamycin (42 ppm in the diet) ad libitum for 8 months. Treatment was started at two different ages to gauge effectiveness of an early‐intervention (started at ~30 weeks old, *N* = 12), as well as a late‐intervention (started at ~72 weeks, *N* = 11–12). At the time of behavioral experiments and tissue collection, mice in the early‐intervention group were ~76 weeks old (*N* = 8–12) and those in the late‐intervention group were ~120 weeks old (*N* = 3–8). Given the additional aging of the late‐intervention group, there was a greater attrition of study animals due to age‐related death compared to the early‐intervention group, and therefore, data could be confounded by survivorship bias.

The starting ages for the early‐ and late‐intervention groups were chosen to resemble the studies conducted by the ITP in which rapamycin treatment was started at either 270 days (38.6 weeks) or 600 days (85.7 weeks) of age, whereby both treatment paradigms provided an overall increase in life span (Harrison et al., 2009). This same diet (42 ppm rapa) was previously shown to inhibit mTORC1 in both male and female HET3 mice at old age (>100 weeks), as measured by a reduction in phosphorylated ribosomal protein S6 (Ser240/244), a substrate of S6 kinase 1, in visceral adipose tissue. It was also found that circulating rapamycin was equivalent between males and females (Harrison et al., 2009). The effectiveness of this treatment in our own study was confirmed by reduced p70S6K phosphorylation in the liver of males in the late‐intervention group (Figure S6a).

**FIGURE 7:**
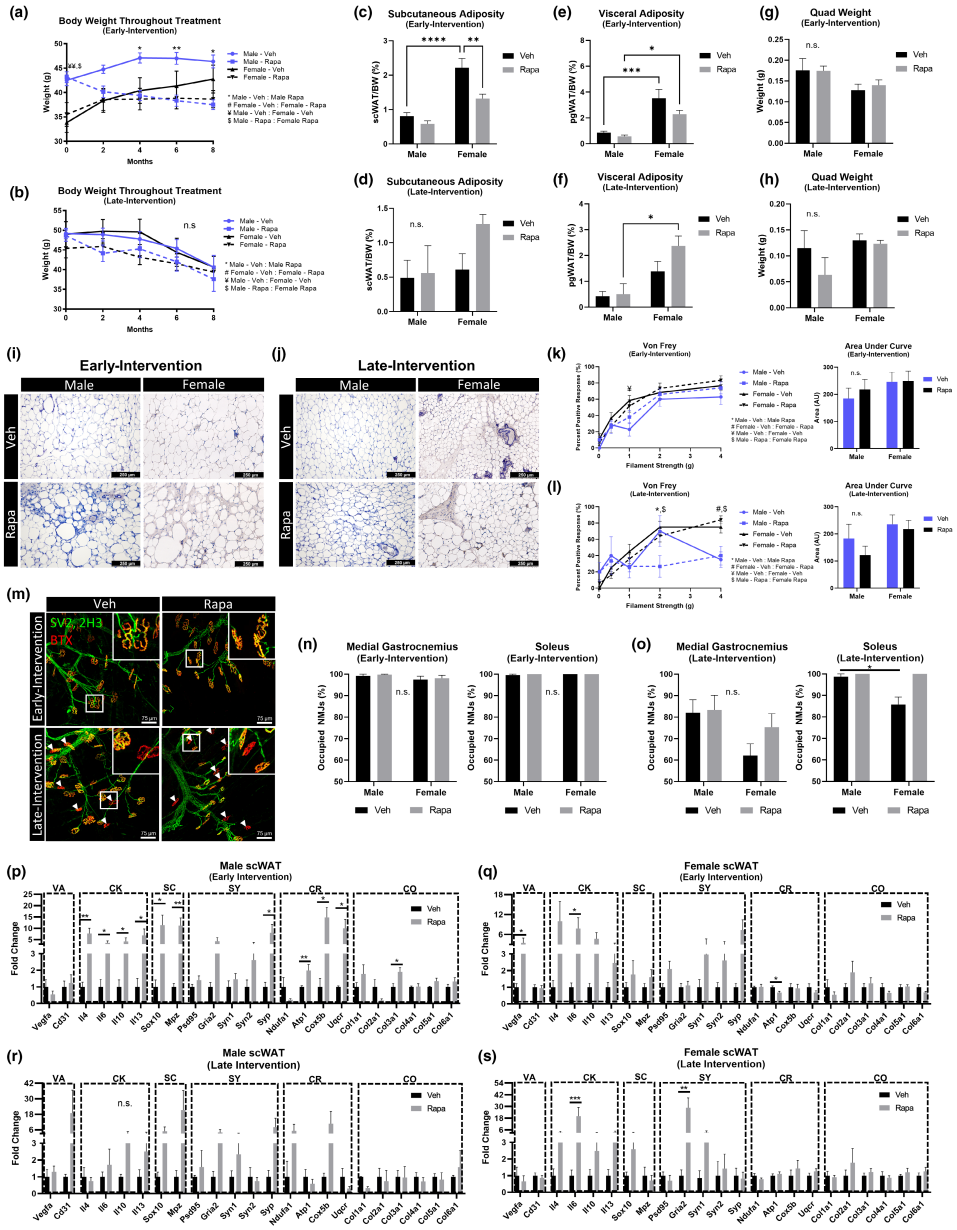
Rapamycin treatment had no effect on age‐related neuropathy in HET3 mice. Male and female HET3 mice were diet‐fed rapamycin (42 ppm) (Rapa) or received standard chow (Veh) for 8 months. Treatment was either started at 30 weeks (early‐intervention) or at 72 weeks (late intervention). Body weights were recorded through duration of treatment (a,b). Subcutaneous (c,d) and visceral (e,f) adiposity, and quad weight (g,h) at time tissue collection. Hematoxylin/Hemalum staining of scWAT (i,j). Von Frey tactile allodynia assay with area under each curve quantified (k,l). Medial gastrocnemius and soleus muscles were stained for NMJ occupation, nerve/pre‐synapse (SV2, 2H3), post‐synapse (BTX). Representative images of medial gastrocnemius muscles (m). White boxes are digital zoom‐ins of NMJs and white arrows mark unoccupied NMJs (m). NMJ occupation was quantified for medial gastrocnemius and soleus for early‐ (n) and late‐intervention groups (o). Gene expression of axillary scWAT was measured by qPCR (p–s). Genes organized into functionally similar groups: vasculature (VA), cytokines (CK), Schwann cell (SC), synaptic (SY), cellular respiration (CR), and collagen (CO). *N* = 3–12. Two‐way ANOVA with Tukey's correction for multiple comparisons (a–h,k,l,n,o). Mann–Whitney nonparametric test (p–s). All error bars are SEMs. ^n.s.^ *p* > 0.05, *p < 0.05, **p < 0.01, ***p < 0.001, ****p < 0.0001. a = male‐veh:male‐rapa; b = female‐veh:female‐rapa; c = male‐veh:female‐veh; d = male‐rapa:female‐rapa

Body weights were measured throughout the 8‐month treatment (Figure 7a,b), and we found that rapamycin decreased body weight in males more than females. This difference was most prominent in the early‐intervention group. Rapamycin treatment decreased subcutaneous adiposity only when started early in life (Figure 7c,d), with no significant changes to visceral adiposity (Figure 7e,f) or quadriceps weight (Figure 7g,h) regardless of when treatment was started. Hematoxylin staining of scWAT sections revealed that rapamycin increased the presence of crown‐like structures, lipofuscin, and likely immune cell infiltration in the early‐intervention group (Figure 7i), but this was not observed in the late‐intervention group which, although older, unexpectedly had fewer signs of inflammation than the rapamycin‐treated mice of the early‐intervention group (Figure 7j).

### 3.8 Diet‐fed rapamycin did not change the peripheral neuropathy phenotype in aged HET3 mice

A von Frey test was used to gauge the level of peripheral neuropathy in the hind paws of HET3 mice following rapamycin treatment. Early‐intervention (Figure 7k) and late‐intervention (Figure 7l) rapamycin treatments had no impact on sensitivity to mechanical stimuli. A previous study using the hot/cold plate nociceptive test also did not find that rapamycin had any impact on paw sensitivity (Neff et al., 2013). NMJ occupation of the medial gastrocnemius (Figure 7n) and soleus remained mostly intact for the early‐intervention group. This is consistent with occupation observed at similar ages in HET3 mice (Figure 2e), and this was not further affected by rapamycin (Figure 7n). Similarly, in the late‐intervention group, and consistent with our other observations (Figure 2e), there was a significant decrease in NMJ occupation due to age which was not mitigated by rapamycin (Figure 7o). Together, these data indicate that rapamycin neither improves nor worsens age‐related peripheral neuropathy.

### 3.9 Diet‐fed rapamycin increased inflammatory, Schwann cell, and synaptic gene expression in scWAT

Males who started rapamycin treatment early in life displayed significant increases in scWAT gene expression for all measured cytokine (*Il4*, *Il6*, *Il10*, *Il13*) and Schwann cell genes (*Sox10*, *Mpz*) (Figure 7q). Synaptic gene expression displayed an overall increase with treatment, with *Syp* being statistically significant (Figure 7p). Mitochondrial genes also increased with rapamycin (*AtP1*, *Cox5b*, and *Atp1* all statistically significant) (Figure 7p). *Col3a1* increased with rapamycin, and no other collagen genes were changed (Figure 7p). Female mice of the early‐intervention group displayed similar gene expression profiles as the males for cytokines, Schwann cells, and synapses; all of which revealed a trend to increase, while collagen genes were relatively unchanged (Figure 7q). Interestingly, females displayed a significant increase in the angiogenic *Vegfa* gene in response to rapamycin, whereas males did not, potentially due to the known link between estrogen action and VEGF expression (Fatima et al., 2017). Mitochondrial gene expression was either unchanged, or significantly decreased (*Atp1*), in the females with rapamycin (Figure 7q). Mice in the late‐intervention group displayed similar overall sex‐specific trends in cytokine, Schwann cell, and synaptic genes, but with fewer gene changes reaching statistical significance (Figure 7r,s). The increased cytokine gene expression could explain the inflammation observed in the male and female early‐intervention group scWAT (Figure 7i), but does not explain why this adipose inflammation appeared diminished or absent in the late‐intervention group (Figure 7j). Additionally, this pro‐inflammatory phenotype in scWAT was likely similar to the reduced life span of rapamycin treated obese C57BL/KsJ*lepr* ^*db/db*^ mice, which was attributed to an increase in inflammation (Sataranatarajan et al., 2016).

### 3.10 Rapamycin treatment started early in life increased scWAT fibrosis

Inhibition of mTORC1 with rapamycin has been used as an effective treatment for TGF‐α‐induced pulmonary fibrosis caused by chronic inflammation (Korfhagen et al., 2009), but is ineffective at treating TGFβ1‐induced pulmonary fibrosis. TGFβ1 activity drives mTORC1 phosphorylation of 4 E‐BP1 and increases ECM distribution, and this phosphorylation by mTORC1 is insensitive to rapamycin (Plate et al., 2020). It was also found that 14 ppm diet‐fed rapamycin treatment caused pancreatic fibrosis in diabetic NONcNZO10/LtJ mice (Reifsnyder et al., 2014). Given our interest in whether rapamycin would be able to ameliorate the WAT fibrosis observed with aging, we stained scWAT from male and female HET3 mice for collagen following early and late‐interventions with rapamycin treatment.

**FIGURE 8:**
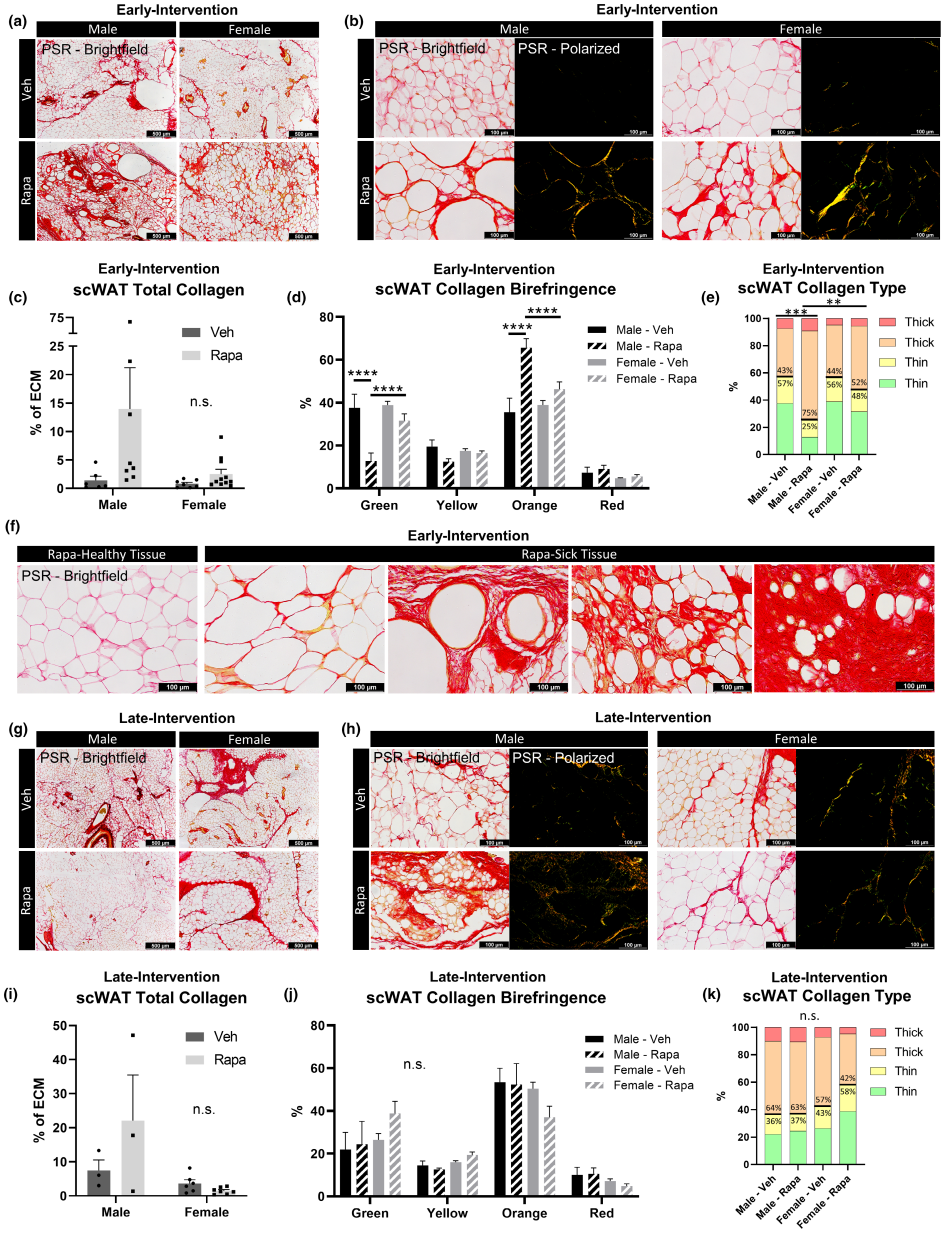
Rapamycin treatment started early in life increased scWAT fibrosis. PSR staining of scWAT from early‐intervention (a–f) versus late‐Intervention (g–k) of rapamycin (42 ppm) treated male and female HET3 mice imaged at 4× objective magnification (a,g). Five representative images of tissue parenchyma were captured separately with bright‐field and polarized light at 20× objective magnification per tissue per animal (b,h). Total collagen was measured as a ratio of birefringent collagen to total PSR staining (c,i). Changes in specific hues of collagen birefringence (d,j). Contribution of collagen fiber thickness determined by hue (e,k). Thin collagen (green and yellow); thick collagen (orange and yellow). *N* = 3–11. Two‐way ANOVA with Tukey's correction for multiple comparisons (c,d,i,j). One‐way ANOVA with Tukey's correction for multiple comparisons (e,k). All error bars are SEMs. ^n.s.^ *p* > 0.05, *p < 0.05, **p < 0.01, ***p < 0.001, ****p < 0.0001

Mice that had been given rapamycin in the early‐intervention group appeared to have greater collagen deposition than the vehicle group (Figure 8a,b), with 3 out of 8 males and 3 out of 11 females exhibiting severely fibrotic tissue, worse than any observations made with aging alone (Figure 4). However, the total increase in collagen was not statistically significant between treatment groups (Figure 8c). Birefringence analysis revealed that in males, the rapamycin decreased green fibers (*p* < 0.0001) and increased orange (*p* < 0.0001), similar to what was observed with aging, but this change was more pronounced in males than females (green, *p* < 0.0001; orange, *p* < 0.0001) (Figure 8d). Males demonstrated a significant increase in thick collagen fibers (orange + red) following rapamycin treatment (Veh, 57%:43%; Rapa, 25%:75%; *p* = 0.0007) (Figure 8e) that was more pronounced than females following rapamycin treatment (*p* = 0.0058) (Figure 8e). Of note, males treated with rapamycin in the early‐intervention group displayed the lowest ratio of thin to thick collagen fibers that we have observed thus far. The striking fibrosis observed in a handful of male and female HET3 mice following rapamycin treatment was displayed by comparing representative images of tissues with the lowest recorded total collagen (“Rapa‐Healthy”), to those tissues with the highest total collagen after rapamycin treatment (“Rapa‐Sick”) (Figure 8f). The exacerbated fibrosis is possibly due to inhibition of mTORC2 as a result of the chronic rapamycin treatment, as mTORC2 regulates cytoskeletal organization (Laplante & Sabatini, 2009). Again, variations within these two subsets of rapamycin‐treated mice may be due to underlying genetic contributions from the founder strains.

Mice (116–120 weeks old) that had been given rapamycin in the late‐intervention group were less susceptible to the fibrosis‐inducing effects of rapamycin treatment. Male mice demonstrated a possible increase in total collagen, whereas the females showed no difference between vehicle and rapamycin treated groups (Figure 8g,h). Differences observed in the males failed to meet statistical significance (Figure 8i). Birefringence patterns did not vary by treatment or sex (Figure 8j) and neither did the ratio of collagen fiber thickness (Figure 8k). These data support the conclusion that treatment with rapamycin at this dose and duration has the potential of being more harmful to adipose tissue health if started early in life rather than late.

## 4 DISCUSSION

### 4.1 Age‐induced changes to adipose tissues

With age and across genetic strains, we observed numerous differences in pathophysiological changes to adipose tissues. Changes to adipose tissue were also often depot‐specific, with an age‐induced redistribution of fat mass that prioritized accumulation of visceral adipose tissue (Palmer & Kirkland, 2016). These changes also varied by sex, as observed previously (Bond et al., 2021; Hoffman & Valencak, 2021). Though a general increase in fat mass occurred with age (as seen in BL6 mice aged to 75 weeks, Figure 1b,c), at later ages (~100 weeks), a decrease in fat mass was observed in BL6 mice (Hemmeryckx et al., 2010). These observations were similar in HET3 mice (Figure 1g,i). Data indicate an age‐related decline in adipose tissue total vascularity (Donato et al., 2014) and vascular function (Islam et al., 2020), which fits with our observations that adipose vessel innervation changes with age in BL6 mice, but this was not observed in HET3 mice despite changes to vascular function with age. Aging adipose tissue is also characterized by chronic inflammation (De Carvalho et al., 2019), and in our studies, this was most prominent in visceral pgWAT for both strains and was only prominent in scWAT following rapamycin treatment. In adipose tissue, chronic inflammation is tightly linked with tissue fibrosis. As fat mass increases, the proximity of each adipocyte to the vasculature decreases, driving tissue hypoxia and an increase in hypoxia inducible factor (HIF)‐1α which induces tissue fibrosis and inflammation (Halberg et al., 2009). This has primarily been observed and described in obese tissues (Crewe et al., 2017), but similar mechanisms likely occur in aging adipose as well (Zhang et al., 2011). The fibrotic and now inflexible EMC applies shear stress to aging adipocytes as they attempt to increase in size. This causes cell damage, lysis, and an increased inflammatory profile (Khan et al., 2009). Subsequently, increased tissue inflammation can also induce tissue fibrosis since many cytokines, including IL‐13, can activate macrophages or fibroblasts (Wynn, 2008) which can secrete collagen (Kendall & Feghali‐Bostwick, 2014). Both BL6 and HET3 mice displayed this inflamed and fibrotic aged adipose phenotype, as demonstrated by histological assessment (Figures 1 and 4), and HET3 mice displayed increased cytokine and collagen gene expression with age (Figure 3). The fibrotic phenotype was most prevalent in scWAT, potentially due to the larger amount of basal collagen that we observed at a younger age. Previous research in mice on a high‐fat diet hinted at the possibility that females could be protected from adipose fibrosis onset due their greater ability to expand adiposity (Wynn, 2008), which likely also occurs with aging. However, this was not observed in our aging HET3 mice, suggesting that this protection is limited to instances of increased energy intake alone or may be impacted by genetic strain.

In addition to displaying increased total collagen deposition, the relative proportions of thin and thick collagen fibers also changed with age. Young mice, regardless of sex and strain, displayed primarily thin collagen fibers, while aged mice showed an increase in thick collagen fibers (Figure 4). While types I, III, and V collagens all constitute the thick fibrils (Spiess & Zorn, 2007), and type I collagen is generally considered to be thicker than type III (Rich & Whittaker, 2005), birefringence of collagen thickness does not necessarily differentiate between these collagen types. The proportion of type I to type III collagen fibers increases in many tissues with age (Mays et al., 1988) and in instances of fibrosis (Ganganna et al., 2012; McKleroy et al., 2013). In some cases, these findings were based on incorrectly classifying thick‐fiber birefringence as exclusively type I collagen (Tzortzaki et al., 2006). Regardless, these published data coupled with our own new findings (Figure 4) support that aging adipose is distinguished by fibrosis that is characterized by the accumulation of thick collagen fibers, which are possibly more restrictive than thin fibers, further driving shear stress on adipocytes and exacerbating secondary inflammation.

### 4.2 Age‐related peripheral neuropathy

We have demonstrated that both male and female HET3 mice become neuropathic with age, findings that are consistent with data from BL6 mice presented here and elsewhere (Valdez et al., 2010; Verdu et al., 2000). Our data support a model in which age‐related peripheral neuropathy has initial onset in the skin at 65 weeks in males (later in females) and gradually proceeds to the muscle at later ages. This is supported by our observations of reduced sensitivity and nerve fiber density in hind paws at 65 weeks (Figure 2), and a later reduction in NMJ occupation at 95 weeks (~98% occupation). However, the onset of adipose neuropathy is unclear. We previously found that by 65 weeks of age, BL6 mice displayed a reduction in total innervation of their scWAT depot that was prominent around tissue vasculature (Blaszkiewicz et al., 2019). A consistent loss of adipose vessel innervation was not observed in HET3 mice (Figure 5 and Figure S4e) possibly due to genetic background. Regardless, the neuropathic phenotype in adipose is far more prevalent in BL6 mice and appears to be blunted by the genetic diversity of the HET3 mice. Why this blunting was observed for neuropathy in scWAT and not in skin or muscle remains unclear and may be due to other complex changes occurring in adipose with aging, or the fact that total innervation density is more easily quantified in skin and muscle.

PSD95 may be a biomarker for aging‐related neuropathy. In male HET3 mice, we first observed signs of neuropathy in the skin at about 65 weeks (IENF and von Frey data, Figure 2). This was accompanied by increased expression of *Psd95* in the flank skin (Figure 3) in the males, indicating that higher *Psd95* reflects a more neuropathic tissue. Females, which we find are at least partially protected from neuropathy (Figure 2) at this same age, showed no difference in flank skin *Psd95* at this age, but a trend for increased levels at 126 weeks when we observed behavioral signs of neuropathy (Figure 4). We did not observe adipose neuropathy in male or female HET3 mice by histology, and similarly, we did not observe a significant increase in scWAT *Psd95* expression in either sex across ages. Rapamycin treatment, which was unable to attenuate any neuropathic phenotypes, also did not alter levels of scWAT *Psd95* expression (Figure 6). *Psd95* is likely upregulated in neuropathic tissues to compensate for the loss of pre‐synaptic nerves and terminals, and this compensation eventually wanes (Figure 4d), or it is shed as a biomarker of peripheral neuropathy, as is seen in other neurodegenerative states like Alzheimer's disease (Kivisäkk et al., 2021). Alternatively, PSD95 may be upregulated as an attempt at nerve recovery and regrowth, as is seen after sciatic nerve crush (Gao et al., 2008). However, the changes in pre‐synaptic gene expression (*Syn1*, *Syn2*, and *Syp*) are less clear‐cut, as changes appear to be sex, age, tissue, and strain specific (Figure 3). Schwann cell gene expression in scWAT decreased in males across aging, but increased in female adipose across aging, and was restored in males by rapamycin treatment, indicating that Schwann cells may be another biomarker for adipose neuropathy but it is unclear why rapamycin changed Schwann cell markers in adipose but did not impact innervation status.

### 4.3 Genetic and sex‐dependent differences

While direct comparisons cannot be made between BL6 and HET3 mice due to differences in ages across experimental cohorts, we noted many adipose tissue similarities between the strains as they aged, which we summarized with available literature from these aged mice in Table S2. At approximately middle age (65–75 weeks old), BL6 and HET3 mice had similar adipose tissue distribution, cell size, inflammation, and fibrosis. At similar ages, both BL6 and HET3 mice also displayed neuropathy in the skin and muscle. The only substantial differences between genetic strains were specific to scWAT, namely gene expression and neurovascular innervation, and these appeared to demonstrate protection in the HET3 model, likely due to the greater genetic diversity in this strain.

By contrast, sexual dimorphism was prominent in the HET3 mice, also summarized in Table S2. Males and females displayed different patterns of adiposity changes across aging, and females had a delayed onset of skin neuropathy (as has previously been observed in female BTBR *ob/ob* mice as well (O'Brien et al., 2015). Additionally, while males had decreased synaptic and Schwann cell gene expression with aging, the females had an increase, potentially conferring a protective benefit. The key age for neuropathy onset in males was 62 weeks, and this was significantly later in females—likely due to reproductive senescence, although the NMJ neuropathy onset occurred at similar ages between the sexes. The striking scWAT and flank skin gene expression differences between males and females possibly related to the metabolic protection female mice display in their adipose depots (Chang et al., 2018) at middle age, which could be mediated in large part by estrogen (Bjune et al., 2022). Additionally, the hormone fluctuations in female mice at middle to late ages (Diaz Brinton, 2012) likely contribute to the patterns of weight gain and may be pivotal in mediating protection from neuropathic phenotypes until late age when female mice lose estrogen.

### 4.4 Effects of rapamycin

Although rapamycin has been demonstrated time and again to be a reliable means of increasing mean life span, there is mixed evidence supporting rapamycin as a means of combating aging‐related health phenotypes. While some data suggest that rapamycin can slow aging deficits in spontaneous activity and various age‐related organ and tissue alterations (Wilkinson et al., 2012), there is also mounting evidence that rapamycin does not extend life span by combatting all the pathologies of the aging phenotype, but instead by suppressing cancers (Ehninger et al., 2014; Neff et al., 2013). The data presented here would support the latter conjecture, as rapamycin had no mitigating effects on age‐related declines in tactile sensitivity, adipose tissue pathology, or NMJ occupation as summarized in Table S2.

While neither early‐ nor late‐intervention with chronic rapamycin provided any benefit to nerve integrity or responsiveness, aside from increasing expression of some Schwann cell and synaptic genes, the early‐intervention treatment proved to have a more detrimental impact on scWAT than the late‐intervention, potentially due to the chronic treatment causing off‐target effects on mTORC2 that led to diabetic complications, as reported previously. Cytokine gene expression increased regardless of sex or age of treatment onset, but histological assessment revealed that the early‐intervention rapamycin group displayed a striking pro‐inflammatory phenotype, coupled with greater thick‐fiber collagen deposition. A subset of both male and female HET3 mice treated with rapamycin in the early‐intervention group displayed aberrant fibrosis and tissue inflammation when compared to the other mice in their group. Potentially the precise genetic contribution of the four inbred founder strains could explain the variation of this phenotype across HET3 individuals. Genetic loci mapping (Lipman et al., 2004) of the HET3 mice with exacerbated collagen deposition could provide important insights to the genetic factors that predispose mice, or even humans, to the “rapa‐sick” scWAT phenotype observed in some mice, but this is beyond the scope of the current study.

When interpreting these results, we emphasize the fact that this study chose to use the highest effective dose of diet‐fed encapsulated rapamycin as previously tested by the ITP, for reasons described above. Chronic treatment of 42 ppm rapamycin, in addition to having the greatest increase in mean lifespan, also has some of the most dramatic negative side effects through chronic off‐target inhibition of mTORC2. Known side effects such as glucose intolerance can be mitigated by decreasing the concentrations of rapamycin (Miller et al., 2014) or by intermittent dosing (Arriola Apelo et al., 2016). Here, we provide evidence that a chronic diet‐fed rapamycin intervention started late in life (~72 weeks) caused less adipose tissue inflammation and fibrosis than an intervention started early in life (~30 weeks), while both interventions increase life span (Harrison et al., 2009), indicating that this may be a preferable treatment approach in order to promote healthy life span.

## 5 CONCLUSIONS

While we observed striking impacts on adipose tissue and peripheral neuropathy pathophysiology with aging, these differed by sex and genetic strain, and were not mitigated by rapamycin longevity treatment. Taken together, we have demonstrated that despite rapamycin's success as a longevity agent in male and female animals, it may be uncoupled from health outcomes, and depending on dose and duration, it may reduce the health of adipose tissue and not mitigate peripheral nervous system degradation with aging.

## AUTHOR CONTRIBUTIONS

JWW designed experiments, analyzed data, and wrote the manuscript. MR performed histology, imaging, and qPCR. GM, SD, and EP performed qPCRs. ZA, SKM, and HC processed and analyzed histology. MB conducted IENF. GG performed WB. BT, AH, LR, and LL performed wire myography studies and PVAT histology and assessment. DEH and PCR provided HET3 mice and aging expertise for data analysis and interpretation. KLT conceptualized and oversaw the project, analyzed data, and wrote the manuscript.

## CONFLICT OF INTEREST

The authors declare no competing interests.

## Supporting information


Figure S1.

Figure S2.

Figure S3.

Figure S4.

Figure S5.

Figure S6.

Table S1.

Table S2.

## Data Availability

The data that support the findings of this study are available from the corresponding author upon reasonable request.
