# Supplementary figures and images for: Age‐related changes to adipose tissue and peripheral neuropathy in genetically diverse HET3 mice differ by sex and are not mitigated by rapamycin longevity treatment
Source: Aging Cell. 2023 Feb 16;22(4):e13784. doi: 10.1111/acel.13784 (PMC10086534; doi:10.1111/acel.13784)

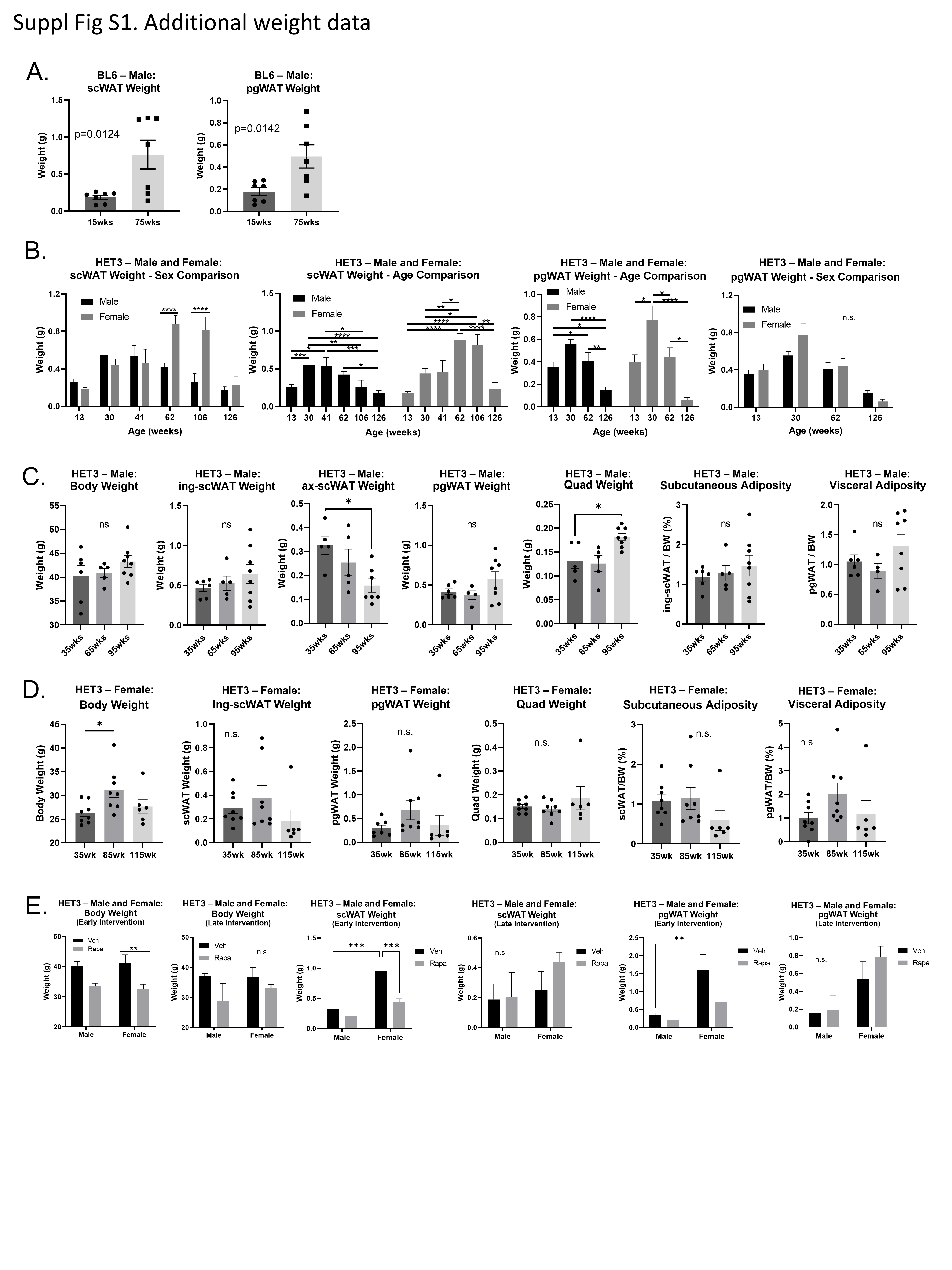

Supplement: Supplementary file 1 — Figure S1. [file ACEL-22-e13784-s003.TIF]

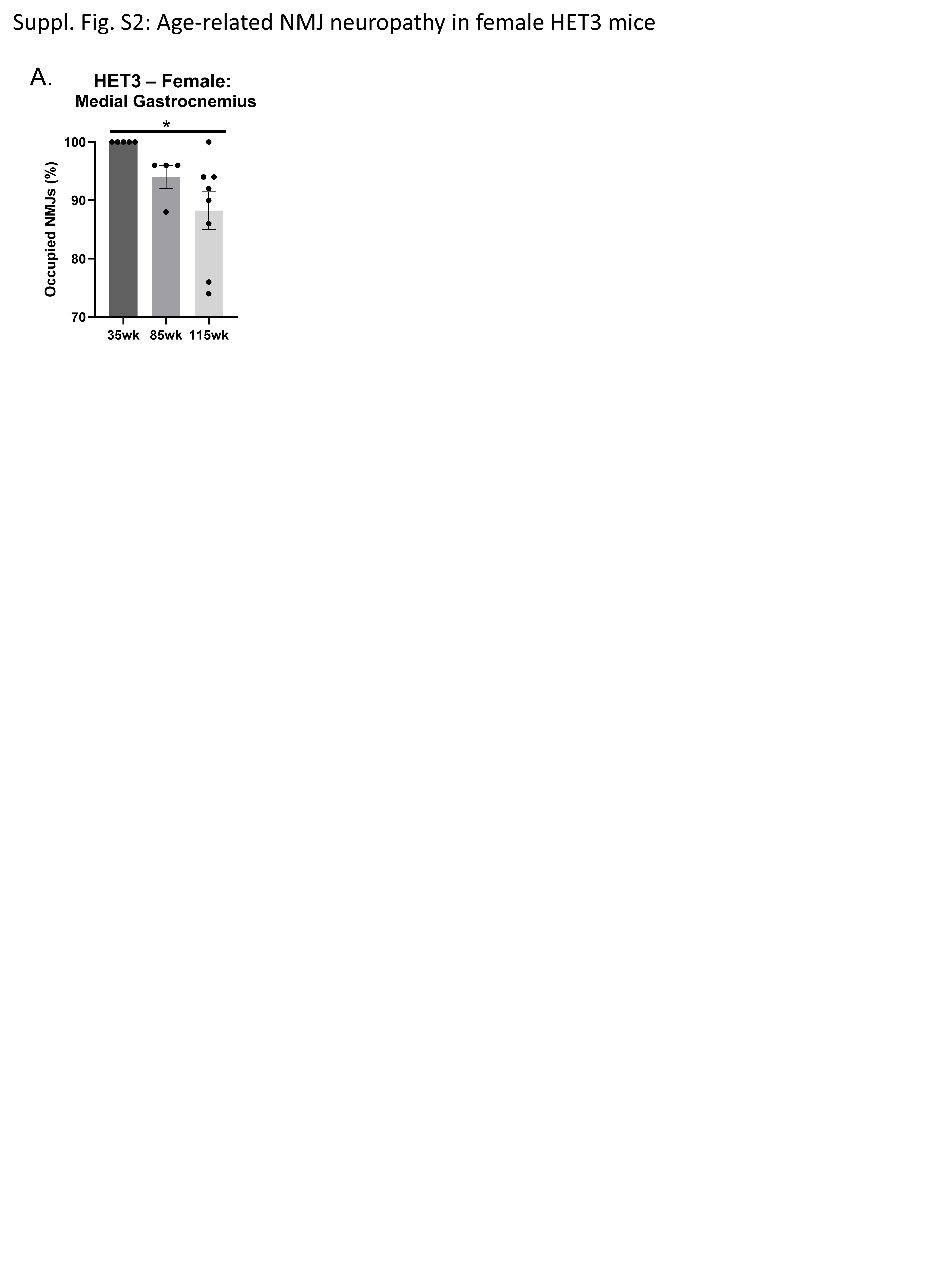

Supplement: Supplementary file 2 — Figure S2. [file ACEL-22-e13784-s002.TIF]

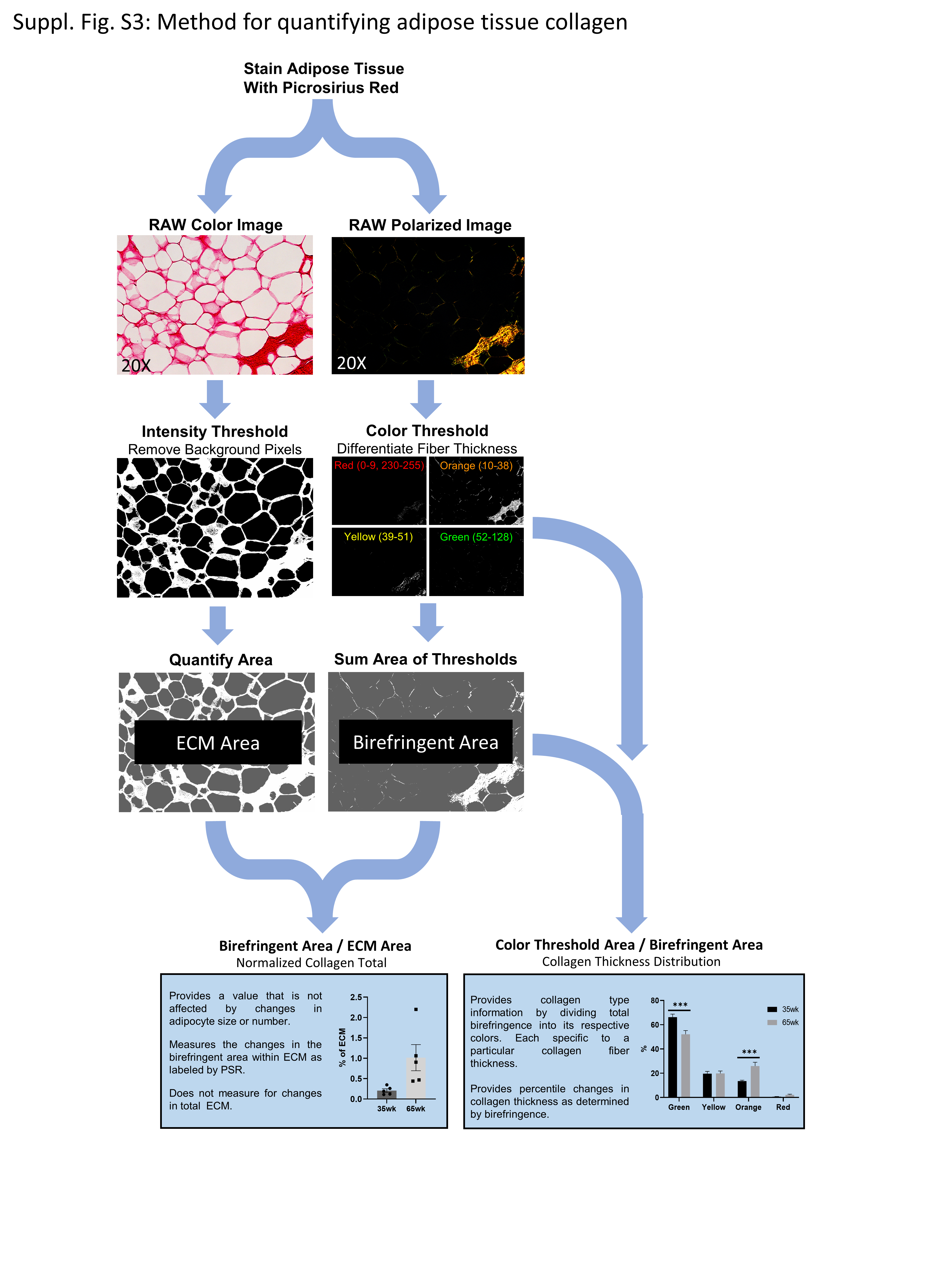

Supplement: Supplementary file 3 — Figure S3. [file ACEL-22-e13784-s005.TIF]

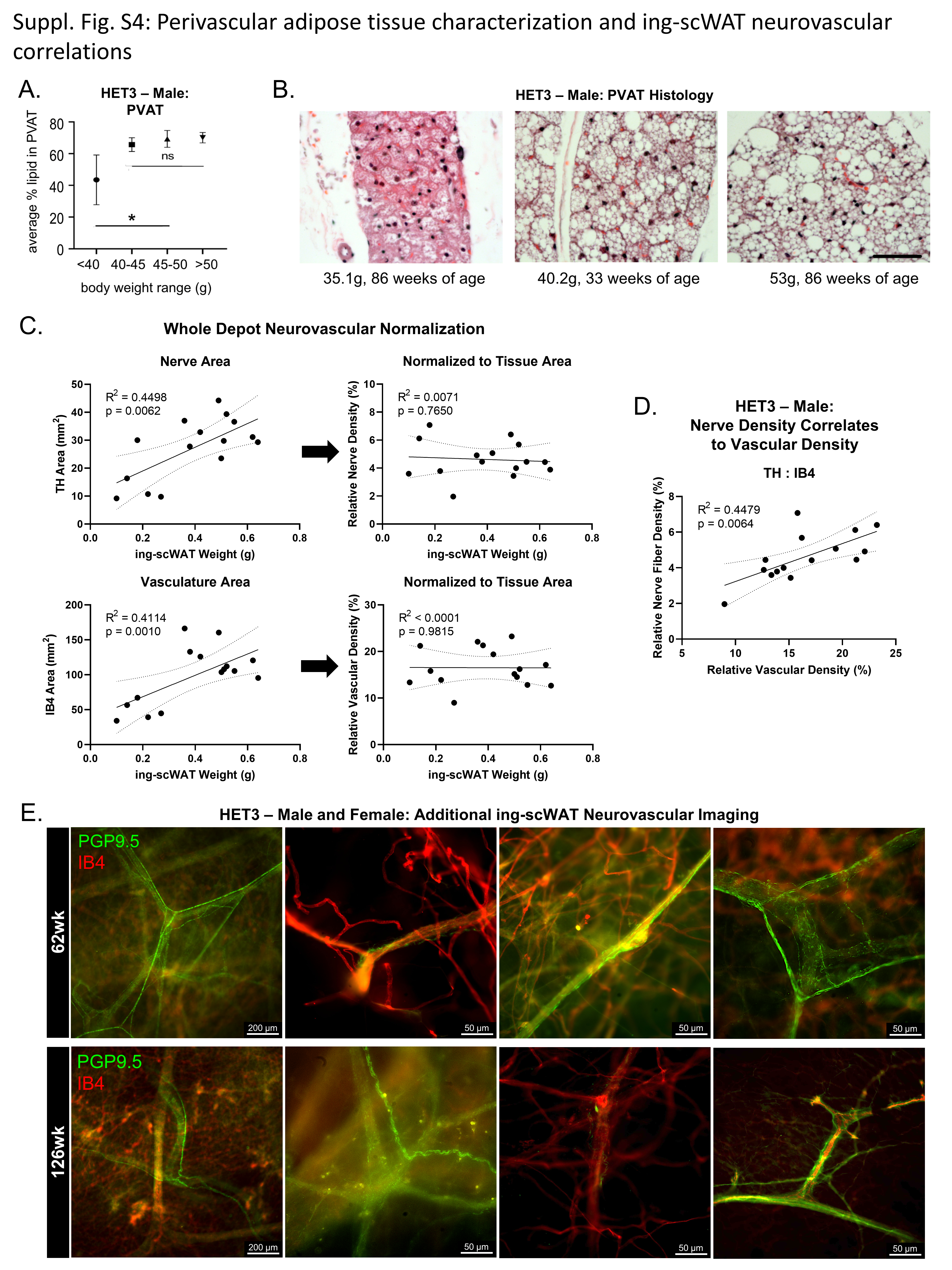

Supplement: Supplementary file 4 — Figure S4. [file ACEL-22-e13784-s006.TIF]

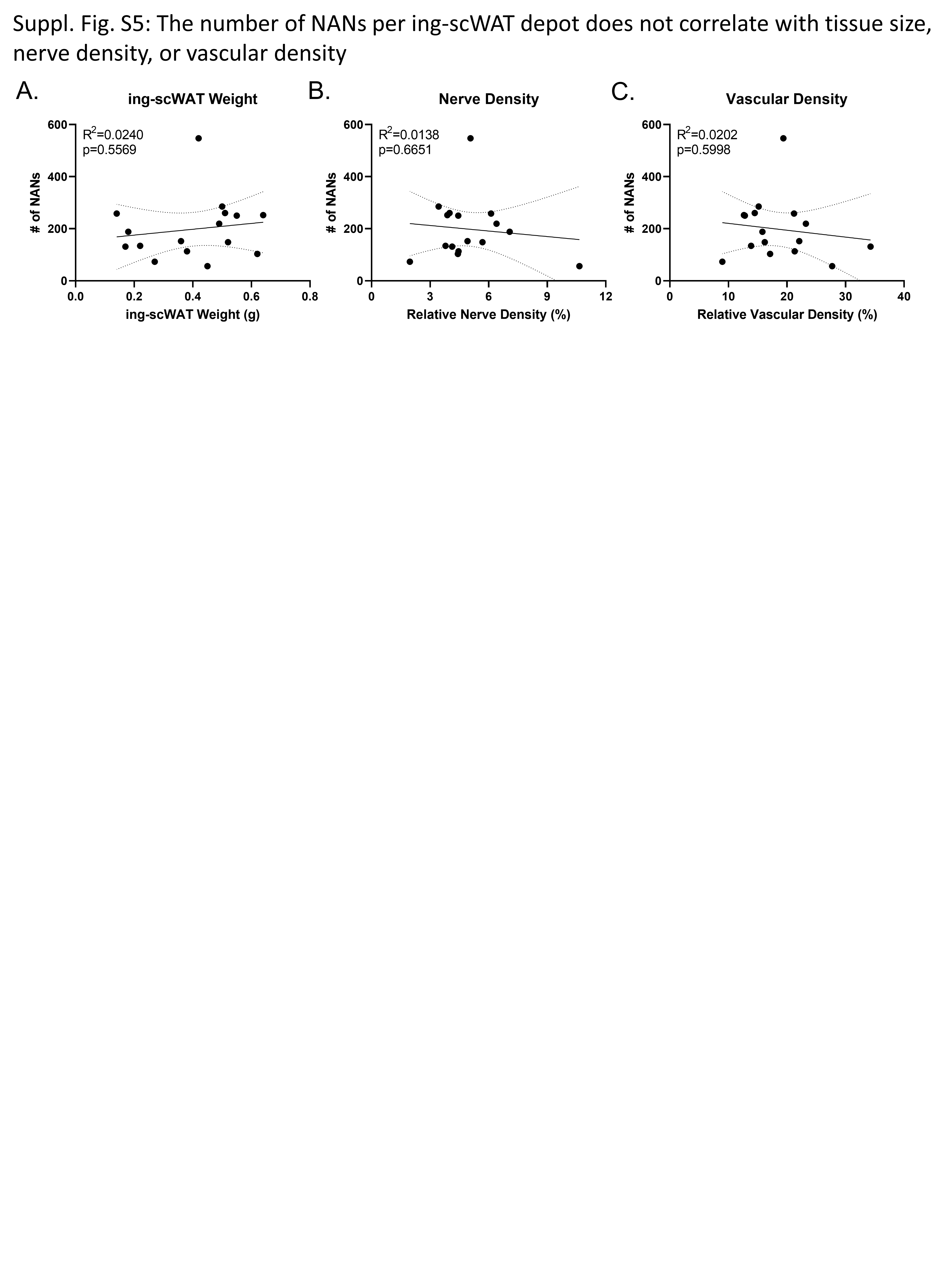

Supplement: Supplementary file 5 — Figure S5. [file ACEL-22-e13784-s008.TIF]

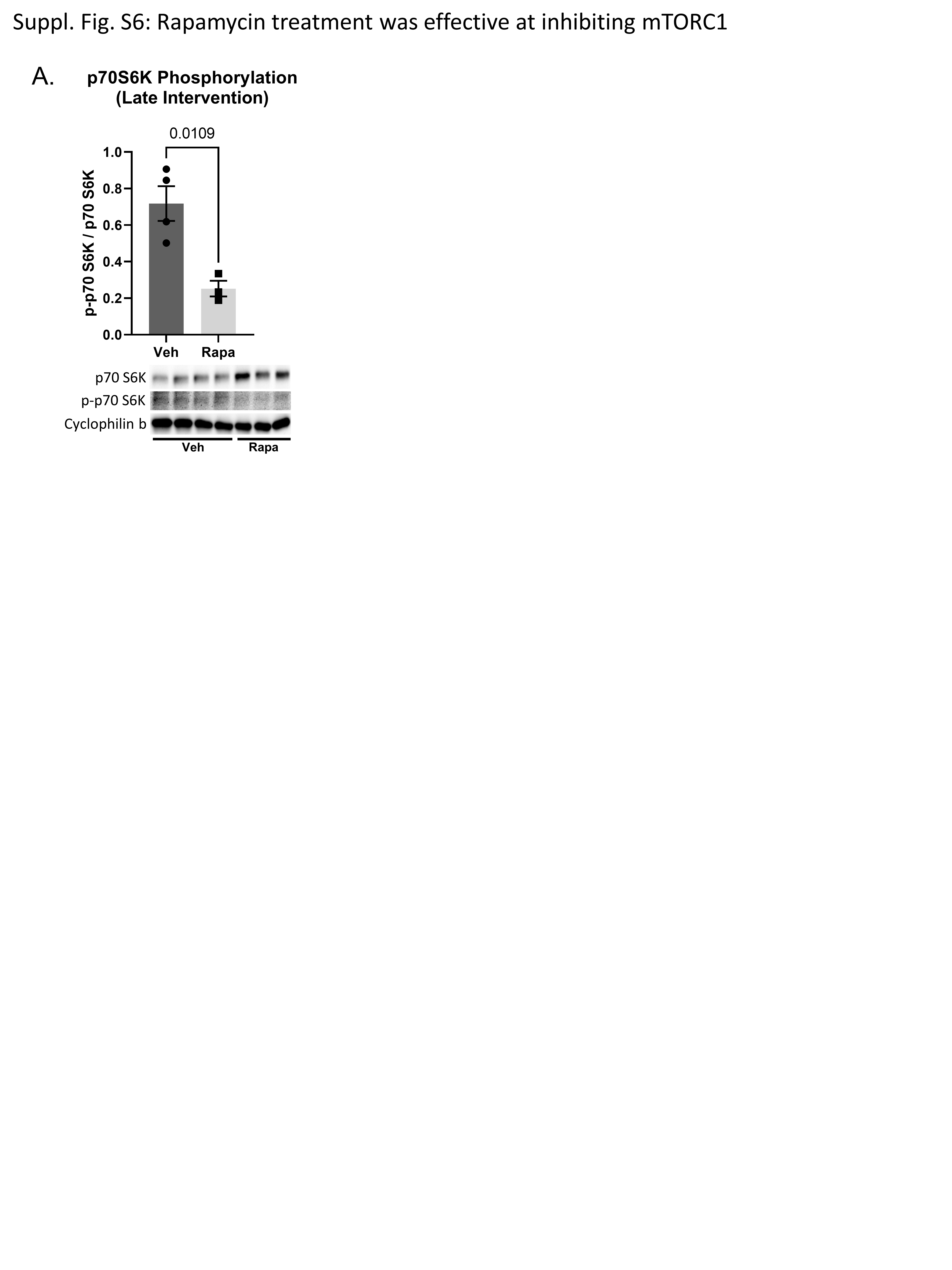

Supplement: Supplementary file 6 — Figure S6. [file ACEL-22-e13784-s001.TIF]

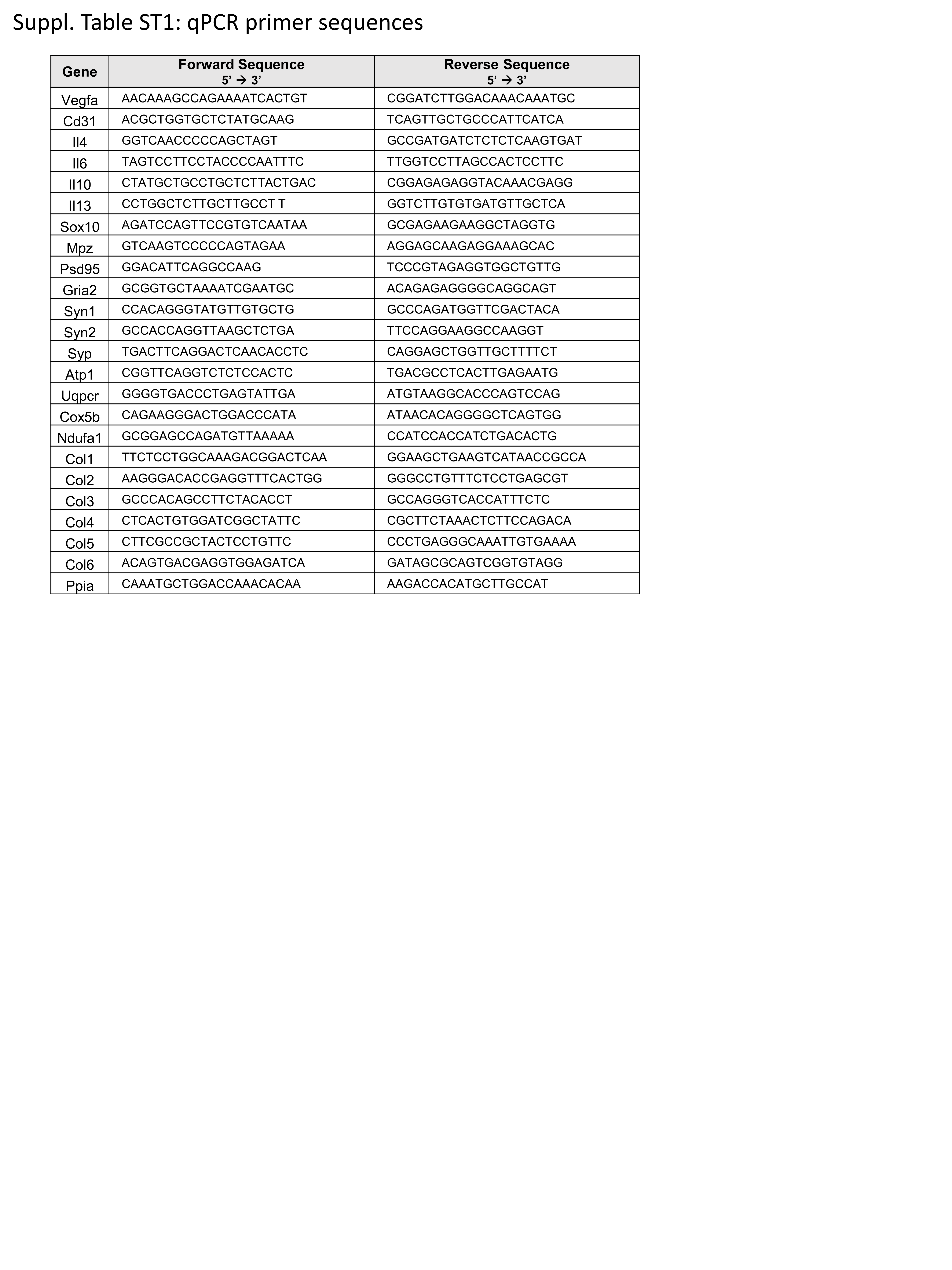

Supplement: Supplementary file 7 — Table S1. [file ACEL-22-e13784-s004.TIF]

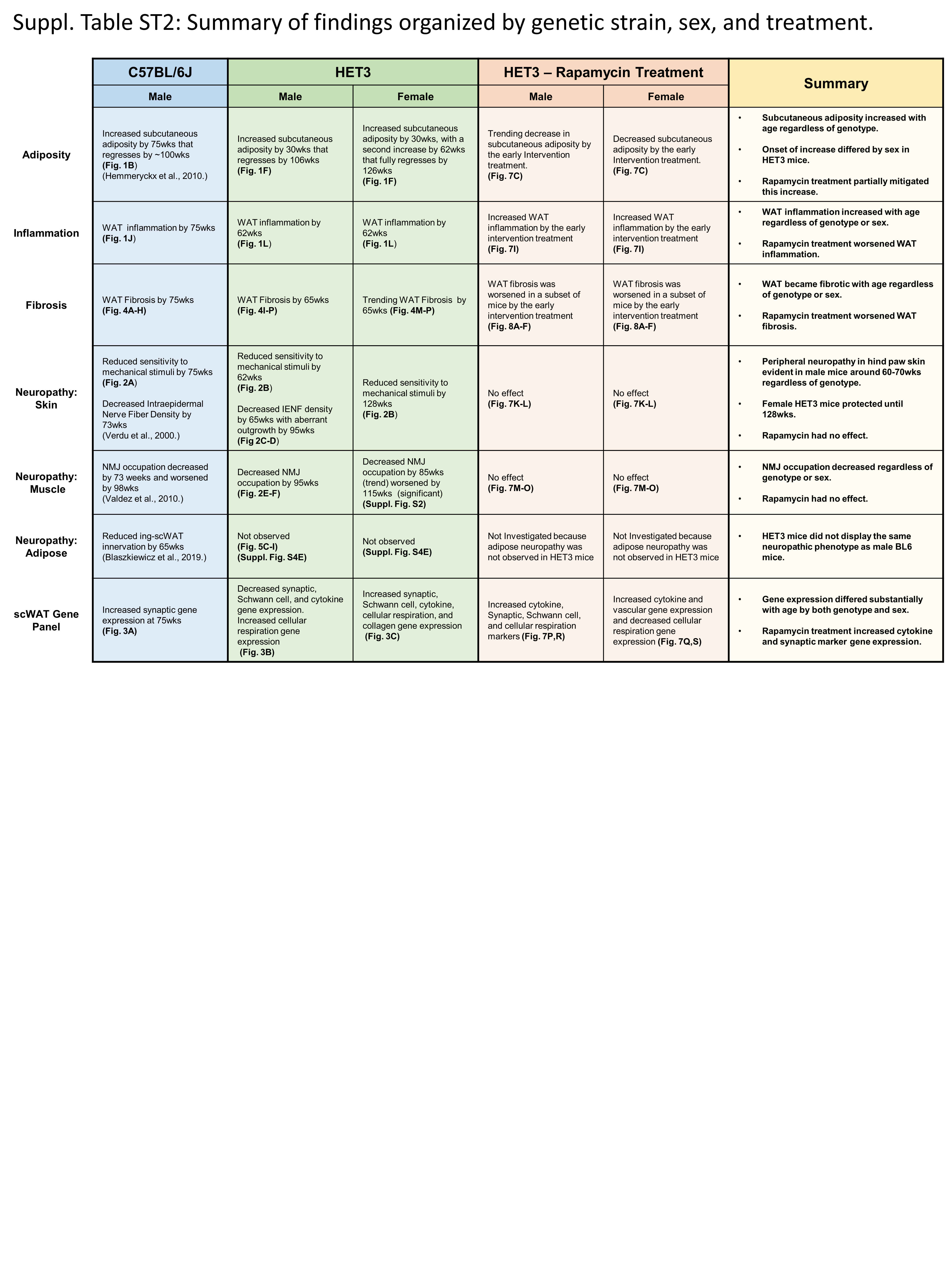

Supplement: Supplementary file 8 — Table S2. [file ACEL-22-e13784-s007.TIF]
